# Mapping and Genetic Structure Analysis of the Anthracnose Resistance Locus *Co-1^HY ^*in the Common Bean (*Phaseolus vulgaris* L.)

**DOI:** 10.1371/journal.pone.0169954

**Published:** 2017-01-11

**Authors:** Mingli Chen, Jing Wu, Lanfen Wang, Nitin Mantri, Xiaoyan Zhang, Zhendong Zhu, Shumin Wang

**Affiliations:** 1 Key Laboratory of Crop Germplasm Resources and Utilization, Ministry of Agriculture; The National Key Facility for Crop Gene Resources and Genetic Improvement, Institute of Crop Science, the Chinese Academy of Agricultural Sciences, Beijing, China; 2 Tobacco Research Institute, Chinese Academy of Agricultural Science, Qingdao, Shandong, China; 3 RMIT University, School of Science, Melbourne, Victoria, Australia; 4 Qingdao Academy of Agricultural Sciences, Shandong, China; Università Politecnica delle Marche, ITALY

## Abstract

Anthracnose is a destructive disease of the common bean (*Phaseolus vulgaris* L.). The Andean cultivar Hongyundou has been demonstrated to possess strong resistance to anthracnose race 81. To study the genetics of this resistance, the Hongyundou cultivar was crossed with a susceptible genotype Jingdou. Segregation of resistance for race 81 was assessed in the F_2_ population and F_2:3_ lines under controlled conditions. Results indicate that Hongyundou carries a single dominant gene for anthracnose resistance. An allele test by crossing Hongyundou with another resistant cultivar revealed that the resistance gene is in the *Co-1* locus (therefore named *Co-1*^*HY*^). The physical distance between this locus and the two flanking markers was 46 kb, and this region included four candidate genes, namely, *Phvul*.*001G243500*, *Phvul*.*001G243600*, *Phvul*.*001G243700* and *Phvul*.*001G243800*. These candidate genes encoded serine/threonine-protein kinases. Expression analysis of the four candidate genes in the resistant and susceptible cultivars under control condition and inoculated treatment revealed that all the four candidate genes are expressed at significantly higher levels in the resistant genotype than in susceptible genotype. *Phvul*.*001G243600* and *Phvul*.*001G243700* are expressed nearly 15-fold and 90-fold higher in the resistant genotype than in the susceptible parent before inoculation, respectively. Four candidate genes will provide useful information for further research into the resistance mechanism of anthracnose in common bean. The closely linked flanking markers identified here may be useful for transferring the resistance allele *Co-1*^*HY*^ from Hongyundou to elite anthracnose susceptible common bean lines.

## Introduction

Common bean (*Phaseolus vulgaris* L.) is one of the most important and widely cultivated grain legumes, in China. Dry bean production in China in 2014 was 1.046 million tons from 936,000 ha harvested area [1]. In recent years, anthracnose caused by *Colletotrichum lindemuthianum* (Sacc. and Magn) Scrib has become a major cause of reduced dry bean production in China. Yield losses due to anthracnose may reach 100% in extreme cases [2]. Identifying anthracnose resistance genes and introgressing them into elite varieties is therefore desired to control anthracnose and reduce crop loss.

Currently, twenty-three related anthracnose resistance genes have been identified by the ‘*Co*’ symbol, including *Co-1* to *Co-7*, *Co-8*, *Co-9* to *Co-15*, *Co-17*, *Co-u* to *Co-z*, and *CoPv09* [3–5]. Among these genes, *Co-1*, *Co-x* and *Co-w* belong to the Andean gene pool, and the others belong to the Mesoamerican gene pool. Different alleles have been described for the *Co-1*, *Co-3* and *Co-4* genes [4]. Specifically, *Co-1* was identified as possessing the *Co-1*, *Co-1*^*2*^, *Co-1*^*3*^, *Co-1*^*4*^, and *Co-1*^*5*^ alleles [6–8]. Most of the anthracnose resistance genes have been located on common bean linkage maps, and flanking markers were determined. Three genes namely, *Co-1*, *Co-x* and *Co-w*, that mapped to linkage group Pv01 [9,10]. Further, *Co-u* mapped on Pv02 [11], and *Co-13* and *Co-17* mapped onto Pv03 [5,12]. The linkage group Pv04 includes six genes, namely: *Co-3*, *Co-9*, *Co-y*, *Co-z*, *Co-10* and *Co-15* [10,13–17]. Additionally, *Co-5*, *Co-6* and *Co-v* were located on the Pv07 [18,19], *Co-4* on the Pv08 linkage group and *Co-2* on the Pv11 [16,20,21]. Recently, Campa et al. identified a new gene *CoPv09* on Pv09 [3].

Genetic mapping analysis of anthracnose resistance genes indicated that some of these *Co* genes are not distributed randomly but tend to exist in complex clusters of race-specific resistance genes. Tight association of many Co-resistance genes have been well established on LGs Pv01, Pv02, Pv04, Pv07 and Pv11 [11,14,18,22,23] and correspond to the named clusters *Co-1*, *Co-u*, *Co-3*, *Co-5* and *Co-2*, respectively.

At the molecular level, most cloned R genes encode proteins containing a nucleotide binding site (NBS) and a C-terminal leucine-rich repeat (LRR) domain. NBS-LRRs (NL) can be divided into two subclasses on the basis of their amino-terminal sequence. One encode an N-terminal domain with Toll/Interleukin-1 Receptor homology (TIR-NB-LRR, TNL), and the other one encode an N-terminal coiled-coil motif (CC-NB-LRR, CNL) [24]. Many clusters of anthracnose resistance genes have been located at the end of the pseudomolecules and correspond to NL clusters by genetic map and/or linked molecular markers. For example, an important number of NL clusters located at the ends of Pv04 and Pv11 containing R specificities against the fungi *C*. *lindemuthianum*, such as *Co-3* and *Co-2*. Another example of large distal R cluster is the multi-resistance I cluster presenting R gene Co-u located at the end of Pv02 containing TNLs sequences. However, some atypical R genes have been observed, including: *Co-1*, *Co-1*^*2*^, *Co-1*^*3*^, *Co-1*^*4*^, *Co-1*^*5*^, *Co-x*, *and Co-w* located at the end of Pv01, and since they are located in a region that does not contain NL sequences [25].

In our previous research, an anthracnose resistance gene *Co-2322* was identified from the common bean cultivar Hongyundou [26]. Based on the mapping of polymorphic markers identified by bulk segregant analysis, this gene maps to chromosome 1 and is flanked by a simple sequence repeat (SSR) marker, C871, and a cleaved amplified polymorphism sequence (CAPS) marker, g1224, at genetic distance of 3.58 cM and 3.81 cM, respectively. The *Co-2322* locus is present in the Adean bean Hongyundou, with wildly planted in China. This locus is very valuable in the breeding of beans, particularly in those countries where race 81 of *C*.*lindemuthianum* predominate. Breeders in these countries should exercise caution in choosing resistance alleles at the *Co-2322* locus, despite allelic differences in reaction to local races. The objectives of the present study were to (1) test allelism between the resistance gene *Co-2322* from Hongyundou and the *Co-1* locus, (2) develop SSR markers for the target region and construct a high-density genetic map of the *Co-2322* region, (3) fine map the *Co-2322* locus using a large F_2_ population from a cross between Hongyundou and Jingdou genotypes, and identify the candidate gene, and (4) identify the sequence polymorphism and expression pattern of the candidate gene.

## Materials and Methods

### Mapping population

Several common bean genotypes were used to analyze the inheritance and independence of the anthracnose resistance gene present in Hongyundou. The bean genotypes Hongyundou, Honghuayundou and Jingdou were obtained from National Gene Bank (China, Beijing). Other bean genotypes namely, G19833, Kaboon, Michigan Dark Red Kidney (MDRK) and Widusa are from the International Center for Tropical Agriculture (CIAT), Cali, Colombia. Hongyundou is a large-seed red Chinese landrace cultivar of Andean origin, and was used as the source of the resistance. Jingdou is middle-seed brown genotype of the Mesoamerican origin, and was used as the susceptible parent. The genome sequence of G19833 was acted as reference genome [27]. Kaboon carries the *Co-1*^*2*^ gene, and Widusa has a unique *Co-1*^*5*^ allele [6].

The anthracnose resistant genotype, Hongyundou, and the susceptible genotype, Jingdou, were crossed to produce F_1_ plants, F_2_ plants, and a F_2:3_ mapping population. A population of 1,092 F_2:3_ families as developed by self-pollination of the F_2_ plants. This population was used to fine map the anthracnose resistance locus *Co-1*^*HY*^ (previously named *Co-2322*). Another cross was made in between two anthracnose resistant parents, Hongyundou and Michigan Dark Red Kidney (MDRK). The segregating F_2_ population of this cross was used to test allelism between the *Co-1*^*HY*^ locus from Hongyundou and *Co-1* from MDRK. The MDRK is a kidney red genotype of Andean origin, and it is thought that the resistance of MDRK to anthracnose (race 73) is conferred by *Co-1* located on Pv01 and that MDRK is highly resistant to race 81 [28].

### *Colletotrichum lindemuthianum* inoculation procedure and disease screening

The *Colletotrichum lindemuthianum* race 81 was isolated from cultivated common bean in China and used to screen for anthracnose resistance in this study [29]. Race 81 was obtained from monosporic cultures maintained on fungus-colonized filter paper at -20°C for long-term storage. To obtain abundant sporulation, race 81 was grown at 19–21°C in darkness for approximately one week on Marthur’s agar [30]. Spore suspensions were prepared by flooding the plates with sterile distilled water and scraping the surface of the culture with a spatula. The two parental cultivars, F_2_ individuals and each of the F_2:3_ families were inoculated with the spore suspension at a final concentration of 2.0×10^6^ spores/ml. Seven- to ten-day-old seedlings were inoculated by spraying with the aqueous conidial suspension and maintained at 20–22% and 95–100% humidity on a 12-h photoperiod in a climate controlled chamber [31]. The disease response of the plants was evaluated after 7–9 days and scored as either resistant (no symptoms) or susceptible (the seedlings died). For each of the F_2:3_ families, at least 30 seedlings were infected and grown as described by Marthur et al. [30], which enabled the classification of the F_2_ plants as homozygous resistant (when all F_3_ individuals were resistant), homozygous susceptible (when all F_3_ individuals were susceptible) or heterozygous (when F_3_ individuals were resistant and susceptible).

### Marker development, bulked segregant analysis using SSRs and genetic mapping

The common bean G19833 reference genome sequence (www.phytozome.org) was used to identify and locate linkage markers. Primer3 software (version 4.0) was used to design PCR primers (http://frodo.wi.mit.edu/primer3/) (S1 Table).

Bulked segregant analysis (BSA) [32] was used to identify genomic SSR markers linked to *Co-2322* (or *Co-1*^*HY*^). Two resistant and two susceptible bulks were prepared for SSR analysis. The resistant and susceptible bulks were obtained by pooling equivalent amounts of DNA from each of the 12 homozygous resistant and 12 homozygous susceptible F_2_ individuals.

Genomic DNA was isolated from lyophilized young leaves using 2×CTAB buffer (2% CTAB, 1.4 M NaCl, 100 mM, pH 8.0; Tris-HCl, 20 mM, pH 8.0 and EDTA) [33].

PCR was performed in a 15 μL mixture containing: 30 ng of DNA or cDNA template, 1.5 μL of 10× *TransTaq* HiFi buffer (including 2.0 mM MgSO_4_), 1.2 μL of 2.5 mM dNTPs, 0.3 μL of each primer at 10 μM and 1.0 U of *TransTaq* HiFi DNA polymerase (TransGen Biotech,China) using the standard PCR program: one cycle of 5 min at 94°C; 35 cycles of 30 s at 94°C, 30 s at 50–58°C, and 1–2 min at 72°C; and a final extension cycle of 10 min at 72°C. The PCR products were resolved on a 6% polyacrylamide gel or a 1% agarose gel. The polymorphic markers were considered putatively linked to the target gene and subsequently confirmed based on their co-segregation patterns in the F_2_ population.

A chi square test was used to test the goodness-of-fit of the observed to the expected ratios in the F_2_ Hongyundou×Jingdou population. The molecular marker data and corresponding individual phenotypes in the F_2:3_ population were analyzed using MAPMAKER Macintosh version 3.0 with a minimum logarithm of odds ratio (LOD) score of 3.0 [34]. The recombination values were converted into genetic map distances (cMs) via the Kosambi mapping function [35].

### Sequence amplification and bioinformatics analysis

Gene-specific primers (S2 Table) were developed and used to amplify the genomic DNA (gDNA), complementary DNA (cDNA) and promoter sequences of the four genes from the resistant and susceptible parents. The *P*.*vulgaris* genome of G19833 genotype was used as the main reference genome [27] for assembly and annotation of candidate genes. Another BAT 93 genotype was used as an additional reference genome [36]. Structural and phylogenetic analysis of the four candidate genes from the common bean genome was performed by comparing them to several known CR4-related protein sequences from *Zea mays* (ZmCR4), *Arabidopsis thaliana* (ACR4 and AtCRR3) and *Oryza sativa* (OsCRR3). The GeneBank protein identification numbers are: ZmCR4, AAB09771.1; ACR4, CAB91612; AtCRR3, CAB87849; OsCRR3, EEE62193. BLAST analysis was used to anchor the mapped markers linked to the *Co-1*^*HY*^ to the reference genomic sequence. The domains were predicted using Pfam 21.0 with an initial E-value cutoff of 0.1 [37]. The sequence alignment and phylogenetic analyses were performed with the DNAStar network service and ClustalW (European Bioinformatics Institute, EBI). Phylogenetic trees were constructed using the neighbor-joining method as implemented in MEGA 6.0 [38,39] with 1,000 bootstrap replicates. A stringency cutoff E value of <E−60 was used to identify similar sequences in other regions of the G19833 genome. The linkage map of homology genes was drawn with MapChart by Voorrips[40]

### Real-time PCR (RT-PCR) analysis

The expression pattern of the four candidate genes, *Phvul*.*001G243500*, *Phvul*.*001G243600*, *Phvul*.*001G243700* and *Phvul*.*001G243800*, in response to inoculation with *C*. *lindemuthianum* race 81 was analyzed in the resistant (Hongyundou) and susceptible (Jingdou) genotypes. A comprehensive temporal expression analysis of the four genes was conducted at 6, 12, 24, 48, 72, 96, and 120 hours after-inoculation (HAI). The leaves from three different treatment and control plants were obtained and snap frozen in liquid nitrogen for RNA isolation. Total RNA was extracted with a GeneJET^TM^ Plant RNA Purification Mini Kit according to the manufacturer’s instructions (Thermo Scientific, USA). cDNA synthesis was performed using a RevertAid^TM^First Strand cDNA Synthesis Kit (Thermo Scientific, USA) using 1 μg of total RNA after DNA enzyme digestion. The candidate gene-specific RT-PCR primers are listed in S2 Table. The primer pair Act-F 5'-GAAGTTCTCTTCCAACCATCC-3' and Act-R 5'-TTTCCTTGCTCATTCTGTCCG-3' was used to amplify the common bean actin gene, which was used as a reference gene. The qPCR was performed in a 20 μL reaction that consisted 2 μL of the cDNA, 0.4 μL each gene-specific forward and reverse primers with concentration 10μM, 10 μL of SYBR *Premix Ex Taq*^TM^, 0.4 μL ROX Reference Dye II (50×), and add distilled water to 20 μL (Takara Bio Inc., Beijing) was run on an ABI 7500 real-time PCR machine (Bio-Rad Corporation, USA) according to the manufacturer’s instructions. The gene expression data from three biological replicates and three technical replicates were processed and standardized according to the comparative 2^-ΔΔCt^ method [41].

## Results

### Inheritance analysis and allele test of anthracnose resistance gene

**Fig 1:**
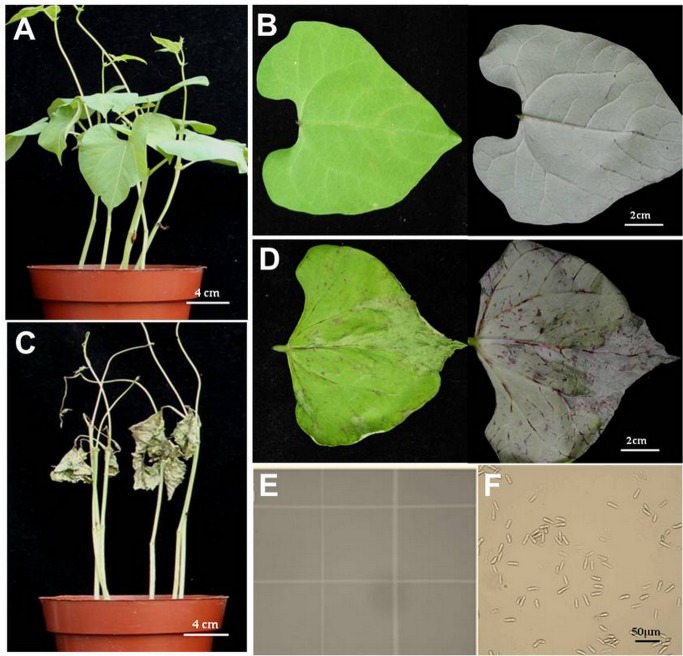
The anthracnose resistant (Hongyundou) and susceptible (Jingdou) plants following *C*. *lindemuthianum* race 81 inoculation. (A) The Hongyundou plants grew normally and were completely asymptomatic. (B) The Jingdou plants began to wilt seven days after inoculation. (C) Sporadic disease spots on the front side and underside of an inoculated Hongyundou leaf. (D) The Jingdou plants exhibited large lesions on the inoculated leaves and collapsed with severe anthracnose symptoms. (E) No spores could be extracted from the inoculated leaves of the Hongyundou plants. (F) Abundant spores were detected from the inoculated leaves of the Jingdou plants.

Seven days post-inoculation, the resistance plants grew normally and with no symptoms or small dark in the leaves (Fig 1A and 1B). However, the susceptible plants were severely wilting and the leaves exhibited largely collapsed lesions (Fig 1C and 1D). Sporulation never occurred from the inoculated leaves of the resistant plants, whereas abundant spores were collected from the inoculated leaves of the susceptible plants (Fig 1E and 1F).

In addition, we tested the reaction of the offspring to races 81. The F_1_ plants exhibited similar phenotype as that of the resistant parent, indicating that the resistance was dominant. Initial analysis of the 182 F_2_ plants identified resistant and susceptible individuals. The segregation of the resistant and susceptible plants was consistent with that expected for segregation at a single locus. The F_2_ population segregated as 3 resistant: 1 susceptible, and the F_2:3_ lines segregated as 247 homozygous resistant:549 segregating:296 homozygous susceptible, which was also expected for single gene segregation (χ^2^_1:2:1_ = 4.43<χ^2^_0.05_ = 5.99; Table 1).

**Table 1:** The anthracnose resistance of the parents and F_2:3_ families.

Parent or cross		No. of plants or lines			Expected ratio	*Χ*^2^	*P-value*
		Resistant	Segregating	Susceptible			
**Hongyundou**	P1	30					
**Jingdou**	P2			30			
**Hongyundou × Jingdou**	F_1_	50					
**Hongyundou × Jingdou**	F_2_	138		44	3:1	0.029	0.864
**Hongyundou × Jingdou**	F_2:3_	247	549	296	1:2:1	4.43	0.109

The values for the significance of the χ^2^ tests at P = 0.05 were 3.83 for 1 df and 5.99 for 2 df.

The resistance gene in Hongyundou was previously mapped onto chromosome 1 [26]. To identify the relationship between *Co-2322* and the *Co-1* locus, an allele test were carried out between the resistance gene in Hongyundou and the *Co-1* locus. The result showed that after inoculation with race 81, the parent plants of Hongyundou and MDRK grew normally without any leaf symptoms, and the 605 F_2_ plants crossed by Hongyundou and MDRK presented the same phenotypic character, remarkable resistance to race 81. This suggests that *Co-2322* may be a member of the *Co-1* allelic series. Therefore, we propose the use of symbol *Co-1*^*HY*^ for the major dominant gene in Hongyundou.

### Identification of markers linked with the *Co-1*^*HY*^ locus

**Fig 2:**
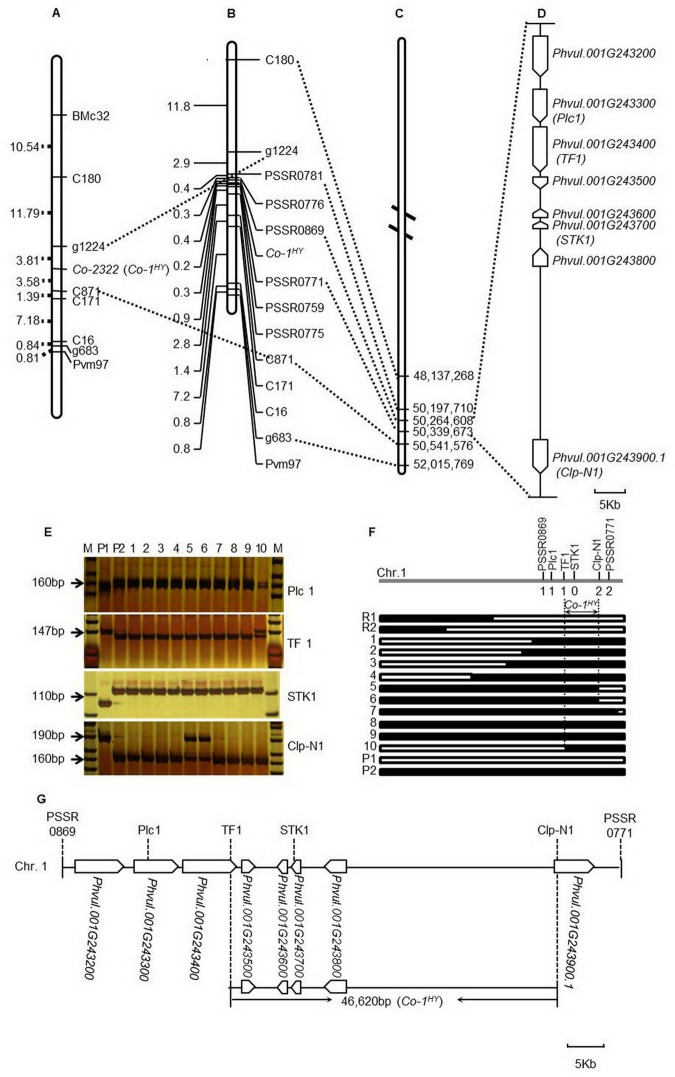
Positional cloning of the resistance gene *Co-1*^*HY*^. (A) The primary genetic linkage map of *Co-2322* gene constructed by Chen et al. [26]. (B) The fine mapping of the *Co-2322* (or *Co-1*^*HY*^) gene and associated molecular markers. Recombination distances are indicated on the left side of the linkage group in centiMorgans (cM), and the marker names are shown on the right side. (C) The physical map showing the locations of the *Co-1*^*HY*^ locus and SSR markers. The *Co-1*^*HY*^ region was positioned between 50,264,608 bp and 50,339,673 bp in an approximately 75 kb interval of chromosome 1 between the SSR markers PSSR0869 and PSSR0771. The dotted lines indicate common markers. (D) The predicted genes flank the target 75-kb region. Fifteen ORFs are shown as open arrows, and the parentheses show PCR markers Plc1, TF1, STK1 and Clp-N1 that were developed with reference to the predicted genes. (E) The amplification of the F_2_ population with the PCR markers Plc1, TF1, STK1 and Clp-N1. P1: resistant parent; P2: susceptible parent; 1–10: susceptible individuals of the F_2_ population; individual 10 in the markers of Plc1 and TF1 and individuals 5 and 6 in Clp-N1 are recombined. (F) Diagrammatic representation of the *Co-1*^*HY*^ mapping. The distribution of recombination events that were detected by high-resolution mapping is depicted. The hatched bar represents the region of the Hongyundou genome, and the open bar represents the region of the Jingdou genome. The number below the markers PSSR0869, Plc1, TF1, STK1, Clp-N1 and PSSR0771 represents the recombinant. Genotyping of the homozygous progeny delimited the *Co-1*^*HY*^ locus to an approximately 46 kb stretch flanked by Clp-N1 and TF1. P1: resistant parent; P2: susceptible parent; 1–10: susceptible individuals of the F_2_ population; R1, R2: resistant individuals of the F_2_ population. (G) Annotation of the 46-kb candidate region. The pentagons represent the predicted genes with the sizes indicated in the lengths of each gene, and the arrows indicate the relative orientation.

In our previous study, we mapped the *Co-1*^*HY*^ to chromosome 1 at a locus flanked by a marker, C871, and a marker, g1224, at genetic distance of 3.58 cM and 3.81 cM, respectively [26] (Fig 2A). The C871 was located at 50,541,576 bp while g1224 could not be mapped, maybe it's because there have some difference between the sequences of g1224 maker and reference genome sequence. Therefore, we selected another linkage marker C180 as an anchor as it was 11.79 cM away from g1224. The C180 marker was anchored at 48,137,268 bp. We then utilized the genome sequence information of the 2.4 Mb region between the nucleotides at 48,137,268 and at 50,541,576. We identified 282 SSR markers from this region that were used to fine map the *Co-1*^*HY*^ locus. Of all of the markers chosen for initial screening, 32 (11.35%) were polymorphic between the parental lines. Ultimately, the genomic SSR markers PSSR0771 and PSSR0869 were polymorphic between the contrasting parents and bulks, which indicated that PSSR0771 and PSSR0869 were tightly linked to the *Co-1*^*HY*^ (Fig 2B). A single recombinant event was detected between PSSR0771 and *Co-1*^*HY*^, whereas two recombinant events were detected between PSSR0869 and *Co-1*^*HY*^. Based on these results, we suggest that *Co-1*^*HY*^ is located between the markers PSSR0771 and PSSR0869. BLAST analysis allowed us to anchor the markers PSSR0771 and PSSR0869 within approximately 75kb region that is located between the 50,339,673 and 50,264,608 bp of the common bean genome (Fig 2C). Subsequently, we used the annotation data of the G19833 reference genome to identify genes located within this 75kb region. We identified eight predicted genes namely, *Phvul*.*001G243200*, *Phvul*.*001G243300*, *Phvul*.*001G243400*, *Phvul*.*001G243500*, *Phvul*.*001G243600*, *Phvul*.*001G243700*, *Phvul*.*001G243800* and *Phvul*.*001G243900* (Fig 2D).

### Fine mapping and physical localization of the *Co-1*^*HY*^ locus

To accurately define the *Co-1*^*HY*^ locus relative to the closely linked flanking markers according to the position on the chromosome, we selected four of the predicted genes (i.e., *Phvul*.*001G243300*, *Phvul*.*001G243400*, *Phvul*.*001G243700* and *Phvul*.*001G243900*) and PCR amplified them from the resistant and susceptible parents using primers based on the 5' UTR and 3' UTR sequences. A comparison of DNA sequences from the resistant (Hongyundou) and susceptible (Jingdou) parents revealed a number of single nucleotide polymorphisms (SNPs) and insertions or deletions (InDels) (S1 Fig). Based on these nucleotide differences in the parents, we developed four InDel markers, namely, *Plc1*, *TF1*, *STK1*, and *Clp-N1* (Fig 2D;S1 Fig; S3 Table). Finally, the four InDel markers were amplified from the mapping population that included 296 homozygous susceptible F_2:3_ families from the 1,092 F_2:3_ lines (Fig 2E).

We detected two recombinants for the *Clp-N1* marker, only one recombinant each for the *TF1* and *Plc1* markers, and no recombinant for the *STK1* marker. These results indicate that *STK1* co-segregated with the target gene *Co-1*^*HY*^. We therefore suggest that the *Co-1*^*HY*^ is located between the *TF1* and *Clp-N1* markers (Fig 2E and 2F), and includes a genomic region that is 46,620 bp long and is located between the 50,286,325 and 50,332,945 bp of the G19833 reference genome. There are four potential genes within this 46,620 bp region (Fig 2G). Using the annotation information of the reference genome from the database, all the four predicted genes namely, *Phvul*.*001G243500*, *Phvul*.*001G243600*, *Phvul*.*001G243700* and *Phvul*.*001G243800* are classified as ‘similar to CRINKLY4 related 3’ from *A*. *thaliana* (S4 Table). These four genes, were considered as the candidate genes for the *Co-1*^*HY*^ locus (Fig 2G).

### Analysis of genetic structure and sequence polymorphism of the four candidate genes

The amplification of the four candidate genes reveals that *Phvul*.*001G243500*, *Phvul*.*001G243600*, *Phvul*.*001G243700* and *Phvul*.*001G243800* were 1,470 bp, 1,107 bp, 1,233 bp and 2,421 bp long, respectively in the resistant genotype Hongyundou, with no intron and kinase catalytic domain. Further BLASTP analysis of the predicted protein sequences of the candidate genes showed *Phvul*.*001G243500*, *Phvul*.*001G243600*, *Phvul*.*001G243700* and *Phvul*.*001G243800* from Hongyundou encoded proteins with 489 AA, 368 AA, 410 AA and 806 AA, respectively. These predicted proteins matched with the entries PHASIBEAM10F002206T1, PHASIBEAM10F002205T1, PHASIBEAM10F002204T1 and PHASIBEAM10F002203T1, respectively from the BAT93 database. All the four predicted proteins shared sequence similarities with *A*. *thaliana CRINKLY4*-related 3 (CRR3, AT3G55950).

Structural and phylogenetic analyses indicated all the four candidate genes were more similar to AtCRR3 (*Arabidopsis thaliana*) than other CR4-related genes such as ZmCR4 (*Zea mays*), ACR4 (*Arabidopsis thaliana*) and OsCRR3 (*Oryza sativa*). The *Phvul*.*001G243800* encodes a full-length AtCRR3 protein with 97% query coverage. It has 58% identity and 71% similarity. Further, the proteins encoded by *Phvul*.*001G243500*, *Phvul*.*001G243600* and *Phvul*.*001G243700* only included the C-terminal part of the kinase, with 38–40% amino acid query coverage, 52%-58% identities and 63%-69% similarities. All of the four predicted proteins lacked crinkly repeats but contained kinase domains (S2 Fig). We further determined the phylogenetic relationships between these four genes. The four proteins fell into 2 major clusters (S3 Fig). *Phvul*.*001G243500*, *Phvul*.*001G243600* and *Phvul*.*001G243700* were more closely related to each other than *Phvul*.*001G243800*. Interestingly, *Phvul*.*001G243800* and AtCRR3 formed a clade and appeared to be potential orthologs.

All the candidate genes had single polymorphic differences in the genomic, cDNA and amino acid sequences in parents. The sequences of *Phvul*.*001G243500*, *Phvul*.*001G243600*, *Phvul*.*001G243700* and *Phvul*.*001G243800* revealed SNPs corresponding to one amino acid variation in *Phvul*.*001G243500* and *Phvul*.*001G243800*, and two amino acid variations in *Phvul*.*001G243600* (S4 Fig). Interestingly, compared to the resistant parent, *Phvul*.*001G243700* contained ten SNPs and three missing nucleotides near the termination codon of the cDNA sequences that resulted in six polymorphic sites and one missing amino acid (arginine) in the susceptible parent (S4 Fig). To corroborate the role of these polymorphisms in resistance or susceptibility to anthracnose, we sequenced the *Phvul*.*001G243700* homologs from four different anthracnose resistant cultivars (G19833, Kaboon, MDRK, Honghuayundou) and one other susceptible cultivar (Widusa). These sequences were aligned to the *Phvul*.*001G243700* homologs from the Hongyundou and Jingdou cultivars. Astonishingly, the *Phvul*.*001G243700* from Kaboon, MDRK, Honghuayundou and Hongyundou were 100% identical, whereas the *Phvul*.*001G243700* from G19833 and Hongyundou were 99% identical. Remarkably, the susceptible cultivars Widusa and Jingdou had same four amino acid sites among the six polymorphic sites (S5 Fig).

To further examine the transcriptional regulation of these four genes, we analyzed the upstream promoter sequences of the genes using the PlantCARE database. The BLAST searches revealed that the amplified sequence included essential *cis*-regulatory elements of the promoters such as the core promoter element TATA and the common element CAAT that were highly conserved across the investigated species (S6 Fig). Moreover, other specific *cis*-acting elements were also detected in all those predicted genes. For example, TC-rich repeats involved in defense and stress responsiveness were detected in *Phvul*.*001G243500*, *Phvul*.*001G243700* and *Phvul*.*001G243800*; a CGTCA motif involved in Me-JA responsiveness was detected in *Phvul*.*001G243600*; and the 5UTR Py-rich stretch element that confers high transcription levels was detected only in *Phvul*.*001G243700*. The *Phvul*.*001G243800* also contained the TA-rich region (enhancer), Box-W1 (the fungal elicitor responsive element), P-box (the gibberellin-responsive element) and others. There were nearly no differences between the promoter sequences of *Phvul*.*001G243500* and *Phvul*.*001G243600* from the Hongyundou and Jingdou genotypes. We obtained about 1.5kb sequence for the *Phvul*.*001G243700* gene. Interestingly, at approximately 600 bp upstream of the start codon, a sequence of approximately 130 bp was deleted from the Hongyundou, whilst at approximately 800 bp upstream of the start codon, there was an approximately 60 bp insertion. Quite remarkably, the 130 bp missing sequence and 60 bp insertion sequence in Hongyundou all include a TATA and CAAT box that are known to be essential transcription regulators in eukaryotes (S6 Fig).

### Homology search in *P*. *vulgaris* genome sequence

**Fig 3:**
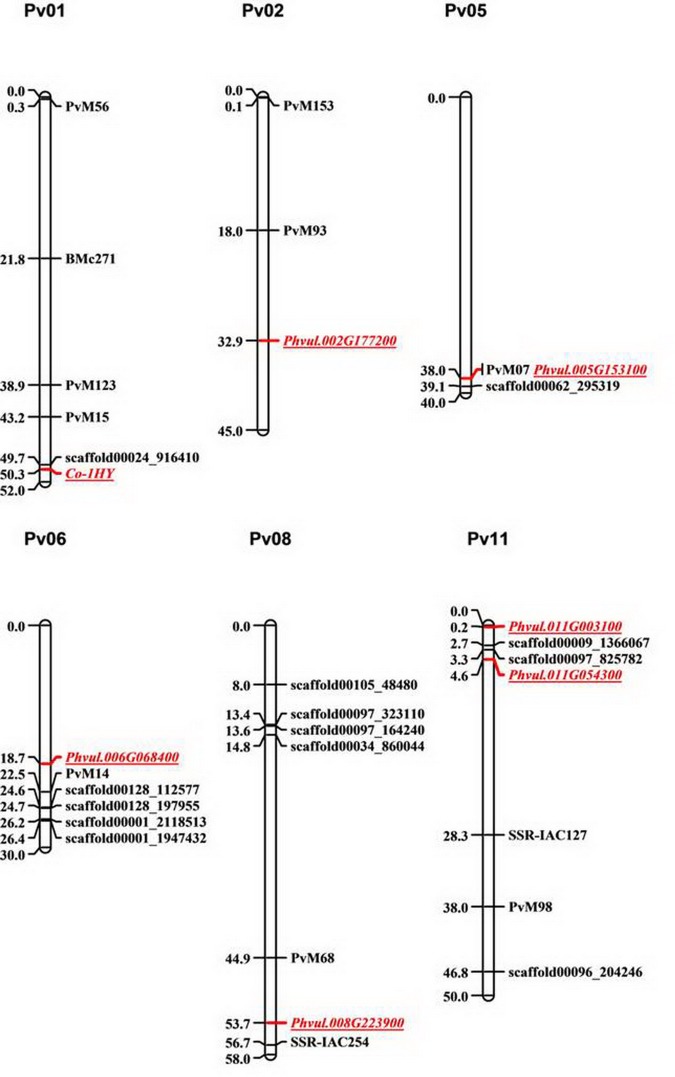
The eight homologous genes were positioned on the common bean linkage map. Representative markers in black are associated with anthracnose resistance and those in red genes homologous to four candidate gene identified from the *Co-1*^*HY*^ region. The marker positions are represented in Mb.

Homologous sequences were accessed of each four candidate gene within the G19833 reference genome (Fig 3). The representative markers by Perseguini are associated with anthracnose resistance [42].In addition to the candidate genes identified in the 46 kb *Co-1*^*HY*^ target region, *Phvul*.*001G243500* identified 5 additional specific genes located on Pv05, Pv06, Pv08 and Pv011 with Kinase domains. These are *Phvul*.*005G153100*, *Phvul*.*006G068400*, *Phvul*.*008G223900*, *Phvul*.*011G003100* and *Phvul*.*011G054300*. Furthermore, *Phvul*.*001G243600* identified 2 homology genes on Pv06 (*Phvul*.*006G068400*) and Pv08 (*Phvul*.*008G223900*). *Phvul*.*001G243700* identified 3 homology genes on Pv06 (*Phvul*.*006G068400*), Pv08 (*Phvul*.*008G223900*) and Pv11 (*Phvul*.*011G003100*) apart from *Co-1*^*HY*^ location. Besides the same location as identified by *Phvul*.*001G243500*, *Phvul*.*001G243800* also had a single homology gene *Phvul*.*002G177200* located on Pv02. However, *Phvul*.*001G243700 and Phvul*.*001G243800* all did not identify sequence similarity to *Phvul*.*001G243500* based on the stringency criteria used. These homology genes can be divided into two categories according to the G19833 annotation. The *Phvul*.*002G177200* gene belongs to the leucine repeat protein kinase (LRR-kinase) family while others have a similar protein domain as the four candidate genes on Pv01. For example, *Phvul*.*005G153100* and *Phvul*.*011G054300* have a CRINKLY 4 (CR4) protein kinase, whilst *Phvul*.*006G068400* and *Phvul*.*008G223900* had a near identical region with CRINKLY 4 related 3 (CRR3) protein, and *Phvul*.*011G003100* had high sequence similarity to CRINKLY 4 related 4 (CRR4) proteins (Table 2). It is noted that the homology genes have characterized by SSR and SNP markers associated with anthracnose (Fig 3).

**Table 2:** Gene homologous with candidate genes *Phvul*.*001G243500*, *Phvul*.*001G243600*, *Phvul*.*001G243700* and *Phvul*.*001G243800*. The G19833 genome sequence was used to identify homologous genes (www.phytozome.org).

Homology gene	G19833 genome location	Number of exon	Coding sequence length (bp)	E-value	Predicted putative annotation
Phvul.002G177200	Chr02:32,856,832..32,862,036	15	2808	7.2E-61	Leucine-rich protein kinase/PTK
Phvul.005G153100	Chr05:38,001,220..38,004,640	1	2721	6.2E-64	CRINKLY4 protein/ CR4
Phvul.006G068400	Chr06:18,689,709..18,692,381	1	2397	0.0	CRINKLY4 Related 3 protein/ CRR3
Phvul.008G223900	Chr08:53,741,844..53,744,583	1	2391	0.0	CRINKLY4 Related 3 protein/ CRR3
Phvul.011G003100	Chr11:237,814..240,513	1	2409	2.5E-128	CRINKLY4 Related 4 protein/ CRR4
Phvul.011G054300	Chr11:4,602,210..4,605,788	1	2760	5.1E-66	CRINKLY4 protein/ CR4

### Expression analysis of the candidate genes

**Fig 4:**
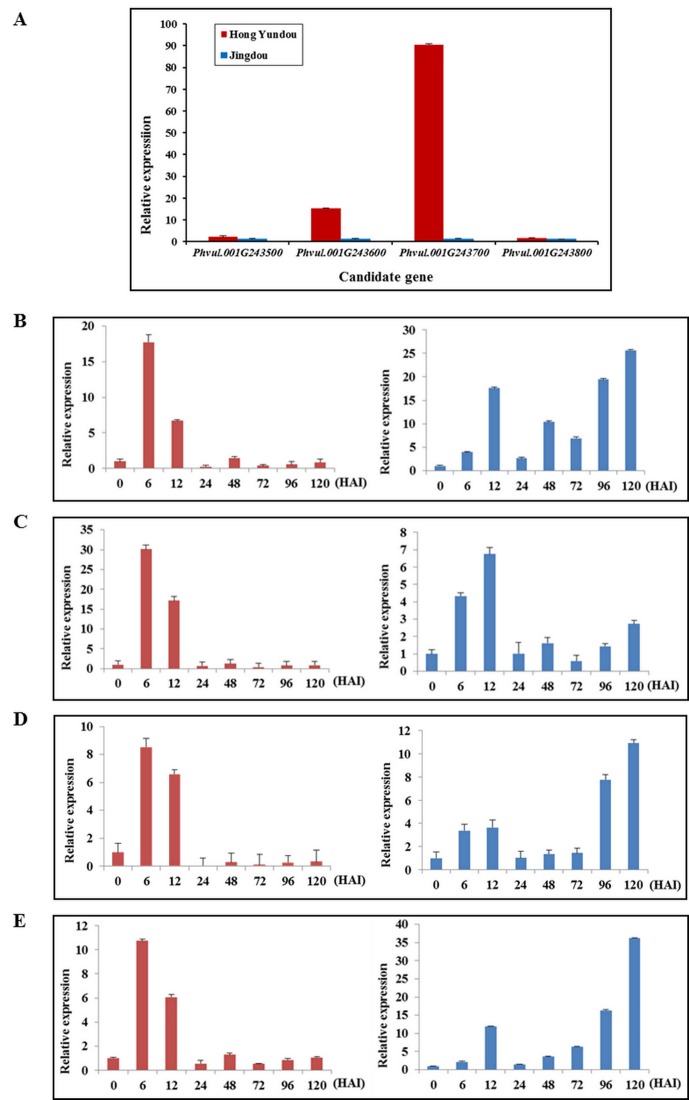
Expression pattern of the four candidate genes with gene-specific primers and the bean actin gene was used as an internal control for qRT-PCR (A) Expression pattern of the four candidate genes in the leaves of the parental Hongyundou and Jingdou plants without inoculation. The graph's horizontal axis indicates the predicted genes (*Phvul*.*001G243500*, *Phvul*.*001G243600*, *Phvul*.*001G243700* and *Phvul*.*001G243800*), and the vertical axis indicates the relative expression levels. (B)-(E) Expression analysis of *Phvul*.*001G243500*, *Phvul*.*001G243600*, *Phvul*.*001G243700* and *Phvul*.*001G243800*, respectively, in the leaves of Hongyundou and Jingdou at different times (0, 6, 12, 24, 48, 72, 96 and 120 hours after inoculation (HAI) with *Colletotrichum lindemuthianum* race 81). Plot with the time after inoculation on the horizontal axis and the relative expression level on the vertical axis. The filled red bars in (A), (B), (C), (D) and (E) refers to Hongyundou, and the blue bars refer to Jingdou. The means were generated from three independent measurements, and the bars indicate the standard errors.

The results revealed that the expression levels of the four genes, i.e., *Phvul*.*001G243500*, *Phvul*.*001G243600*, *Phvul*.*001G243700* and *Phvul*.*001G243800* were higher in the resistant parent than in the susceptible parent by 2.0, 15, 90, and 1.6 fold, respectively (Fig 4A). To further ascertain the roles of these candidate genes in anthracnose resistance, we preformed expression analyses of these genes in the leaves of the resistant and susceptible parents at ternate compound leaf stage, using qRT-PCR with gene-specific primers. Strikingly, temporal gene expression analysis showed that the four candidate genes were induced in anthracnose race 81 infected Hongyundou and Jingdou plants. Interestingly, the expression pattern of the four genes was almost consistently different between resistant and susceptible genotypes. In the resistant genotype, four genes were significantly induced early at 6 HAI, followed by slight reduction in expression at 12 HAI (Fig 4B–4E). Thereafter the expression of these genes in the resistant genotype dropped significantly to almost no noticeable expression from 24 to 120 HAI (Fig 4B–4E). Comparatively, the expression of *Phvul*.*001G243600* peaked at 12 HAI in the susceptible genotype, whilst the expression of other 3 candidates peaked only at 120 HAI. Further, an apparent pattern of some expression at 6 HAI, slightly increased expression at 12 HAI, significantly reduced expression between 24–72 HAI, followed by significantly increased expression at 96 and 120 HAI was observed in the susceptible genotype (Fig 4B–4E). This indicates that the resistant genotype responds very quickly with expression of the four genes peaking at 6 HAI whereas the susceptible genotype with early expression at 6 HAI, manages to lift expression somewhat at 12 HAI, but gives up between 24–72 hours, only to increase expression at a very later stage (96–120 HAI). This suggests that the susceptible genotype misses the critical window of early expression (0–6 HAI), which possibly allows the fungi to penetrate the leaves and may be even multiply within the host (as suggested by increased defense response at later time points). However, low/insignificant gene expression is observed in the susceptible genotype between 24–72 HAI.

## Discussion

Our results reveal that *Co-1*^*HY*^ is a potential member of the *Co-1* allelic series [6,8]. So far, seven alleles related to anthracnose resistance have been reported from the *Co-1* locus on chromosome 1. They are *Co-1* in MDRK, *Co-1*^*2*^ in Kaboon, *Co-1*^*3*^ in Perry Marrow [8], *Co-1*^*4*^ in AND277 [13], *Co-1*^*5*^ in Widusa [6], and *Co-w* and *Co-x* in JaloEEP558 [9]. Here, the allelism test for independence of the single dominant resistant gene in Hongyundou was performed on a single population Hongyundou×MDRK with one race (race81). Moreover, the Widusa (*Co-1*^*5*^), after inoculation with *Colletotrichum lindemuthianum*, was highly susceptible to race 81, which indicates that the resistant gene in Hongyundou has a different allele from *Co-1*^*5*^. This is consistent with previous reports that *Co-1*^*5*^ is a special gene that is different from other reported alleles on the *Co-1* locus [6]. However, further analysis is required to determine if the resistance gene in Hongyundou is different from other alleles that have been previously reported for the *Co-1* locus (example, *Co-1*^*2*^, *Co-1*^*3*^ and *Co-1*^*4*^). Nonetheless, the results support the presence, in Hongyundou, of a resistance allele at the *Co-1* locus.

To date, no anthracnose resistant gene has been cloned from common bean, several complex disease resistance gene clusters have been mapped on the specific linkage that contain classical nucleotide-binding site leucine-rich repeat (*NBS-LRR*) sequence and protein kinases such as *Co-2*, *Co-3*, *Co-4*, *Co-9*, *Co-10*, *Co-y* and *Co-z* [11,43,44,45]. The *Co*-*u* has been localized to the vicinity of TNL sequences [46]. However, fine mapping of the common bean *Co-1*^*HY*^ locus reveals a resistance candidate that encodes a subfamily of *CR4* (*CRR3*) protein, which belong to RLK family. In plant, RLKs are reported to involve in plant defense responses [47,48],such as, *Xa21* and *Xa3* in rice [49,50], *Rpg1 in barley* [51], *RFo1* and *FLS2 in Arabidopsis* [52,53]. However, there are no reports documenting the seemingly direct relevance of the *CR4* to the resistance to anthracnose or other diseases in plants. Previous studies reported that *cr4* gene regulated leaf epidermal cell differentiation and aleurone endosperm cell specialization, AtCRR1, AtCRR2, AtCRR3, and AtCRK1, were identified to play important roles in the protodermal cells of the embryos and shoots and *OsCR4* affect rice seeds in terms of the functions of the palea and lemma during epidermal cell differentiation. [54,55,56,57]. In this paper, the expression level of *Phvul*.*001G243600* and *Phvul*.*001G243700* in the resistant genotype were higher than in the susceptible genotype. Notably, there have some difference in the sequence of these genes between the resistant and susceptible genotype. We also noticed that *Phvul*.*001G243600* contained a CGTCA motif involved in Me-JA response. As Me-JA response is critical to plant defense response [58], the early and significant induction in the resistant genotype along with 2 amino acid difference, makes it a more likely candidate.

Our results suggested that the candidate genes *Phvul*.*001G243500*, *Phvul*.*001G243600*, *Phvul*.*001G243700* and *Phvul*.*001G243800* maybe have new functions. Here, the four candidate genes related to *Co-1*^*HY*^ were more similar to *AtCRR3* than to the other *CR4* members based on the phylogenetic analysis. These findings suggest that the *CRR3* protein may have significant role in plant disease resistance, at least in the common bean. However, Richard et al. [59] noted that *Co-x* is located in the region flanked by two markers, P05 and K06, which are located at nucleotide positions 50,264,307–50,265,284 and 50,320,965–50,322,583 of chromosome 1, respectively. However, the *Co-1*^*HY*^ gene was located between the markers *TF1* and *Clp-N1* with nucleotide position of 50,286,325 and 50,332,737, respectively. These results indicate that the *Co-x* and *Co-1*^*HY*^ genes are likely located in the same locus. In the *Co-x* region, three phosphoinositide-specific phospholipases C (PI-PlC), one zinc finger protein, and four kinases were predicted to be present [59]. The authors speculated the three PI-PLC members as potential *Co-x* candidate genes. Our results reveal that four kinases *Phvul*.*001G243500*, *Phvul*.*001G243600*, *Phvul*.*001G243700*, and *Phvul*.*001G243700* may be more likely the potential *Co-1*^*HY*^ candidate genes. Further studies using for example, a knock-out approach like virus-induced gene silencing are required to ascertain which one of these genes is most important for anthracnose resistance [60].

## Supporting Information

S1 Fig Alignment of the partial nucleotide sequences containing the regions used to develop the PCR markers from the four predicted genes. The white rectangle indicates the insertion deletion length polymorphism.
(TIF)

S2 Fig ClustalX analysis of the candidate genes and several CRINKLY4-related proteins from *Zea mays*, *Arabidopsis thaliana* and *Oryza sativa*. The protein sequences of the candidate gens were amplified from Hongyundou. The following are indicated with symbols and shadings: identical (black shading), 80% identity (grey shading) and 60% identity (light grey shading) residues; the seven ‘crinkly’ repeats (solid bars); the predicted transmembrane domain of ZmCR4 (dotted line labeled TM); and the twelve conserved kinase subdomains (solid bars with round ends).
(PDF)

S3 Fig Phylogenetic tree of the candidate genes *Phvul*.*001G243500*, *Phvul*.*001G243600*, *Phvul*.*001G243700* and *Phvul*.*001G243800*. The utilized multiple sequence alignment is the same as that in A. The results are displayed graphically using NJ-plot from MEGA version 6.0 [38,39]. The bootstrap values from 1,000 bootstrap replicates that were used to assess the robustness of the tree are shown at the nodes.
(TIF)

S4 Fig Alignments of the amino acid sequences of the candidate genes in the resistant (Hongyundou) and susceptible (Jingdou) parents. The black shading indicates polymorphic amino acids.
(PDF)

S5 Fig cDNA sequences *Phvul*.*001G243700* showing single nucleotide polymorphisms (SNPs) (see the rectangle boxes) among seven cultivars. Resistance cultivars: a) G19833, b) Kaboon, c) MDRK, d) Honghuayundou, e) Hongyundou. Susceptible cultivars: f) Jingdou and g) Widusa. Rectangle boxes in solid line reveal the efficient SNPs between resistance cultivars and susceptible cultivars while boxes in dotted line present the meaningless SNPs.
(PDF)

S6 Fig Alignments of the promoter sequences of the candidate genes from the resistant (Hongyundou) and susceptible (Jingdou) parents. The following elements are marked: initiation codon ATG (solid-line arrows); TATA box (grey shading); CAAT box (light grey shading); TC-rich repeats (green shading); TCA-element (red shading); TC-rich repeats (olive green shading); BoxW1 (blue shading); skn-1 motif (blue ash shading); and P-box (clear blue shading).
(PDF)

S1 Table Newly developed genomic SSR markers for the common bean chromosome 1. (DOCX)

S2 Table The primer sequences of amplifying the gDNA, cDNA and promoter sequences of the candidate genes. (DOCX)

S3 Table The primer sequences of the PCR markers. (DOCX)

S4 Table Gene annotation of the target region. (DOCX)
