# Supplementary figures and images for: Mapping and Genetic Structure Analysis of the Anthracnose Resistance Locus Co-1HY in the Common Bean (Phaseolus vulgaris L.)
Source: PLoS One. 2017 Jan 11;12(1):e0169954. doi: 10.1371/journal.pone.0169954 (PMC5226810; doi:10.1371/journal.pone.0169954)

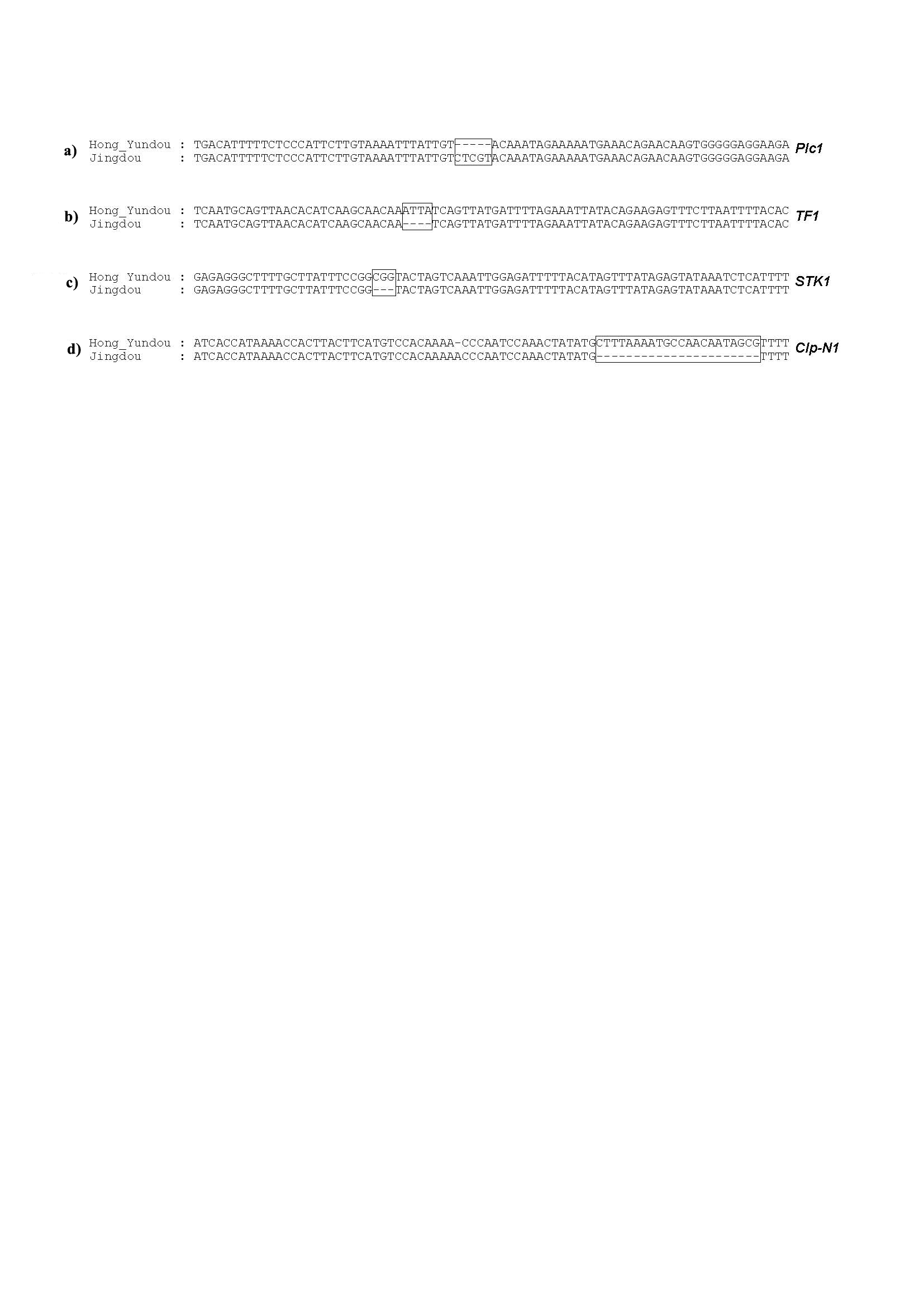

Supplement: S1 Fig — The white rectangle indicates the insertion deletion length polymorphism. (TIF) [file pone.0169954.s001.tif]

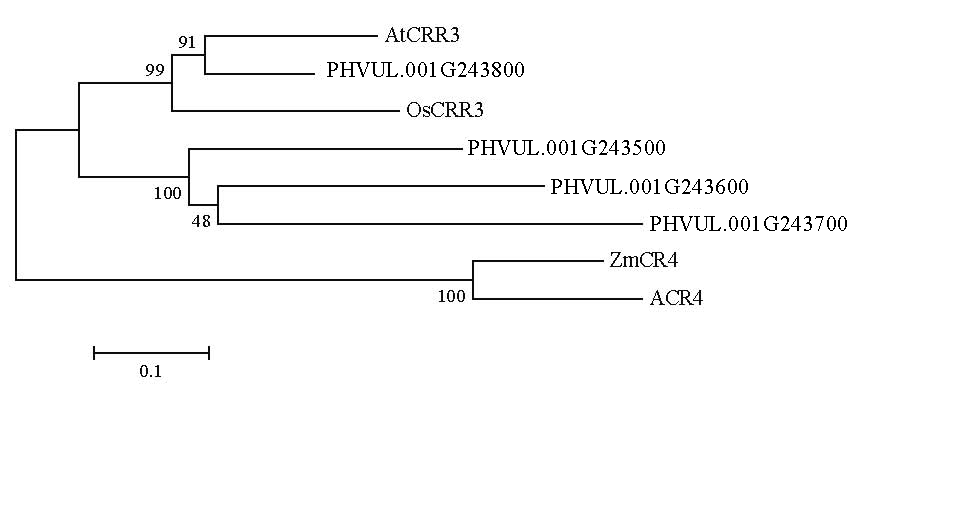

Supplement: S3 Fig — The utilized multiple sequence alignment is the same as that in A. The results are displayed graphically using NJ-plot from MEGA version 6.0 [38,39]. The bootstrap values from 1,000 bootstrap replicates that were used to assess the robustness of the tree are shown at the nodes. (TIF) [file pone.0169954.s003.tif]
