# Supplementary material for: Mapping and Genetic Structure Analysis of the Anthracnose Resistance Locus Co-1HY in the Common Bean (Phaseolus vulgaris L.)
Source: PLoS One. 2017 Jan 11;12(1):e0169954. doi: 10.1371/journal.pone.0169954 (PMC5226810; doi:10.1371/journal.pone.0169954)
Supplement: S2 Fig — The protein sequences of the candidate gens were amplified from Hongyundou. The following are indicated with symbols and shadings: identical (black shading), 80% identity (grey shading) and 60% identity (light grey shading) residues; the seven ‘crinkly’ repeats (solid bars); the predicted transmembrane domain of ZmCR4 (dotted line labeled TM); and the twelve conserved kinase subdomains (solid bars with round ends). (PDF) [file pone.0169954.s002.pdf]

```
                                                                    I
ZmCR4               : M...DHVPA...LVLAGCCFLALLPGWACGLGSMSSIAVSYGEDGPVFCGLNSDGSHLVACFGADASVLY :  64
ACR4                : M.RMFETRAREWILLVKLVLFTSIWQLASALGSMSSIAISYGEGGSVFCGLKSDGSHLVVCYGSNSAILY :  69
OsCRR3              : M..............TPLLLLLLLPLLLLAPASASTVAIAAG.PTACAVAEGNSTVYCASAT.NSSSSAA :  54
AtCRR3              : MKRFINSTVTFSVTVTIAVIIFFLLSPVTSLGSGSTYAVVYGSDTVCALISGQPTQRILCYDTRLNINVT :  70
PHVUL.001G243800    : MKTLTSSSVTLCL.VVAALLILSLPRSSHGLGSGATLTISDASSTVCAVVARESTRRIECY..RQGQIAS :  67
PHVUL.001G243700    : M..................................................................... :   1
PHVUL.001G243500    : M..................................................................... :   1
PHVUL.001G243600    : M..................................................................... :   1

                                          II
ZmCR4               : GAPPNIPFLGLTAGDGFVCGLLLDTRQPYCWGSNSYVKSGVPQP.MVEGARYSELSAGDNHLCALRAAQD : 133
ACR4                : GTPGHLQFIGLTGGDGFMCGLLMLSHQPYCWGNSAFIQMGVPQP.MTKGAEYLEVSAGDYHLCGLRKPIV : 138
OsCRR3              : VAPFVSFSQVSGGGGAFVCGLQVGGRALFCWPAAAPGQ..LRRVYNGP.GQLSQLAVGGGHVAAYDAA.. : 119
AtCRR3              : LNPGVSFSSIAAGD.NFLCGIRSGGYSLLCWDNIGSYSPNRKRIYQNDNVLLETLSVGDKQICATVNG.. : 137
PHVUL.001G243800    : ITPNASFSTISGGR.NYFCGLRSSNSDLLCWNTSSSFE..RRRLYNDSSVPLENLAVGDTHLCATEVG.. : 132
PHVUL.001G243700    : ...................................................................... :   -
PHVUL.001G243500    : ...................................................................... :   -
PHVUL.001G243600    : ...................................................................... :   -

                                 III                                IV
ZmCR4               : GGRGSS..AATSLIDCWGYNMTATHAVDEAVSTVSAGSVFNCGLFARNRTVFCWGDETVSGVVGLAPR.. : 199
ACR4                : GRRKNSNIISSSLVDCWGYNMTRNFVFDKQLHSLSAGSEFNCALSSKDKSVFCWGDENSSQVISLIPK.. : 206
OsCRR3              : .........ARVIRWWRGGDRFPLWFGGG.FASLVSGDDFTCAVETSTSAVRCWGP.....RGGAVEAGF : 174
AtCRR3              : .........TNSLKCWRGSVSDQSKPPNERFRSISSGVGFSCGVSIRNNRILCWGTDPVK..SNQIQTGF : 196
PHVUL.001G243800    : .........DGAVKCWRTGDTFQLPSATDKFASISSGTGFSCGILKNSYRVRCWGDTSVSDLTERIESVF : 193
PHVUL.001G243700    : ...................................................................... :   -
PHVUL.001G243500    : ...................................................................... :   -
PHVUL.001G243600    : ...................................................................... :   -

                                V                                             VI
ZmCR4               : .DLHFQSIGAGGYHVCGVLEN..AQVFCWGRSLEMQQVVPSSAIGDGDVNIVPMDAMSTVVGGRFHACGI : 266
ACR4                : .EKKFQKIAAGGYHVCGILDGLESRVLCWGKSLEFEEEVTGTSTEEKILDLPPKEPLLAVVGGKFYACGI : 275
OsCRR3              : LNASVSALAAGGSRACGVRRNDGGVLCSGGGVLAPRE......DLYVDGLA.........VGDSHACGL : 228
AtCRR3              : GNTPMVTISAGKSHACGLN.TTGNLICIGNNDSGQLNVIAPDQPNLYSSSLS.........LGSNFTCAM : 256
PHVUL.001G243800    : GNMSMLSLVAGGSHVCGLN.STGFLVCGGNNDSGQRD..FPQGGAFEYSGLA.........LGAEHGCAI : 251
PHVUL.001G243700    : .....................................................................A :   2
PHVUL.001G243500    : ...................................................................... :   -
PHVUL.001G243600    : ...................................................................... :   -

                                                        VII
ZmCR4               : RSLDHQVACWGFTLHNSTSPPKG.LKMYALVAGDYFTCGVPAETSLMPRCWGNSGPLALPMAVPPGICVP : 335
ACR4                : KRYDHSAVCWGFFVNRSTPAPTG.IGFYDLAAGNYFTCGVLTGTSMSPVCWGLGFPASIPLAVSPGLCID : 344
OsCRR3              : LRPNHTAACWSLGGATTTLYYPAVGTAFELLVAGGNLTCGLVSANFSLLCWSR..DGLVAAEVNLPPILP : 296
AtCRR3              : RISNNSVVCWGG.GAERFNNVTDS.ISFESISSGPGLICGLISSNLSIMCWNP..TNFSRIFLPFPEVLP : 322
PHVUL.001G243800    : RGLNGSVVCWGGNGSFSVNNVTEG.VSFEVIVSGSNFVCGLTTNNLKVVCWGPGWSNSSTFELPFSTVLP : 320
PHVUL.001G243700    : NSGASLVHSFASIVEYAIRSIGGDLGAFPLRDFSFAVENAWSS.IGGDLGAVP........VRGFASIVE :  63
PHVUL.001G243500    : ........DSESVESIEMWNYG....SFDAFSFSLAVDNEIRSGTSSNLGASSP.......LHSFASLLD :  52
PHVUL.001G243600    : ..................ANN.....AVDARSFSWAVDSAIASESASNLGESR........VHSFASVVD :  40

ZmCR4               : TACSHGYYEYVNHGEVGSIKVCKPANSRLCLPCSTGCPEGLYESSPCNATADRVCQFDCLKCVTDECLSF : 405
ACR4                : TPCPPGTHELSNQENS....PCKFTGSHICLPCSTSCPPGMYQKSVCTERSDQVCVYNCSSCSSHDCSSN : 410
OsCRR3              : GVCVSDNSS.......CKCGPLPDSGRFCKVSG....DVICRRFCDTSPP.....PPP..PSPRTPSPPA : 348
AtCRR3              : GPCVESSSSSL.....CSCGVYPQSDKLCSGTGSICK..SCPIQFPASPPSQFPLPPP..PPPPPPSPST : 383
PHVUL.001G243800    : GSCVQSS.........CECGSYLDSQSLCSGSGNICKPMTCKLQTTAPPPPSLSPPPPSMSPPPPPPPPP : 381
PHVUL.001G243700    : NMMRSVGGD............................................................. :  72
PHVUL.001G243500    : IAIRSRTSSN.......WETSSANSYAFGLDNAIRSRTSSNLETSSAYSFAFGVDTAIRSRTSSNWETSS : 115
PHVUL.001G243600    : GAIRSSTAS............................................................. :  49

                                       TM
                                  ....................
ZmCR4               : CLSQKRTKSR...KLMAFQMRIFVAEIVFAVVLVLSVSVTTCLYVRHKLRHCQCSNRELRLAKSTAYSFR : 472
ACR4                : CSSSATSGGKEKGKFWSLQLPIATAEIGFALFLVAVVSITAALYIRYRLRNCRCSENDTRSSKDSA..FT : 478
OsCRR3              : TPSSRRGVSKGWIAFAVVGAVGCFAGLCSIVYCLLFGFCS.HKKVHNSVQPNIASNNNGGGGGAAAAVG : 417
AtCRR3              : SSPPSKALTRGLLAFAIVGSVGAFAGICSVVYCLWTGVCLGKKKVHNSVQPTITRGGSNSR.....SNSS : 448
PHVUL.001G243800    : SSSRSKTLTNGLLAFAIIGSVGAFAGICTIVYCLWSGVCFGKKKVHSSVQPTITRGSSGSNGGGASNNSN : 451
PHVUL.001G243700    : ..............LRALPVHGFASAVENAMIS..IGGDLGVSPMQGFASRVENAMSSIGG......... : 117
PHVUL.001G243500    : THSFAFEEDRTPSNLGSFRVPSFSSGVDYVIRSSSGGTDSETSPVHSFAFAAGASQMAA........... : 174
PHVUL.001G243600    : ....................................EADLEAFPTHYFAFASAWDKRTV........... :  72
```

```
                                                                                 I
ZmCR4             : KDNMK........................IQPDMEDLKIRR.AQEFSYEELEQATGGFSEDSQVG.KGSF : 516
ACR4              : KDNGK........................IRPDLDELQKRRRARVFTYEELEKAADGFKEESIVG.KGSF : 523
OsCRR3            : SGAPSPYGSPNGSLGRLRRQLS..RVMTRQRSGPSSFKDP..AEEFTFAQLAAATKDFAAEAKIG.EGSF : 482
AtCRR3            : NSRSL..........SIRRQGSRMLSMRRQRSGTSSMKHADKAEEFSFSELASATGNFSLENKIG.SGSF : 507
PHVUL.001G243800  : SSISS..........MIMRQTS..MIMRRQRSGTSSTKHPDRAEEFTLAELVAATNNFLLENKIG.AGSF : 508
PHVUL.001G243700  : ...............................DLGTSHSPVHGFQVFTLLELAAATNNFSVDNKIR.AGSS : 155
PHVUL.001G243500  : .................................NVTGHVAKGLQLFSREELVAATNNFSLHNKIG.VGSF : 210
PHVUL.001G243600  : .................................AAR........LFTLAELKAATNNFSIHNKIFCAGSI : 101

                                   II              III         IV
ZmCR4             : SCVFKGILRDGTVVAVKRAI.....KASDVKKSSKEFHNELDLLSRLNHAHLLNLLGYCEDGSERLLVYE : 581
ACR4              : SCVYKGVLRDGTTVAVKRAI.....MSSDKQKNSNEFRTELDLLSRLNHAHLLSLLGYCEECGERLLVYE : 588
OsCRR3            : GTVYRGKLPDGREVAIKRGE..SGPRARKFQEKETAFRSELAFLSRLHHKHLVGFVGYCEESDERLLVYE : 550
AtCRR3            : GVVYRGKLNDGREVAIKRGE..VNAKMKKFQEKETAFDSEIAFLSRLHHKHLVRLVGYCEEREEKLLVYD : 575
PHVUL.001G243800  : GVVYKGKLADGREVAIKRGE..TGSKMKKFQEKESAFESELAFLSRLHHKHLVGLVGFCEEKDERLLVYE : 576
PHVUL.001G243700  : SVVYRGKLVDGSEVTIERVERWSSRTVEEAFWWR..RTSSLKILPGLRPKNLVGLVGLCEEKNERVLVYE : 223
PHVUL.001G243500  : GVVYGGLLVDGREVAIKRNE..TSPKMKEFQEIV..FGYLVTFLGRLHHEQLVGLVGFCEEEDEKLLVYE : 276
PHVUL.001G243600  : SVVHRGKLFDGRQVAVKRAE..ISSKMKEFQER...FGYLWTLLLRLHHKHLVGLVGFCKDKDKRLLVYE : 166

                          V                   VIA                     VIB
ZmCR4             : FMAHGSLYQHLHGKDPNLKKRLN......WARRVTIAVQAARGIEYLHGYACPPVIHRDIKSSNILIDED : 645
ACR4              : FMAHGSLHNHLHGKNKALKEQLD......WVKRVTIAVQAARGIEYLHGYACPPVIHRDIKSSNILIDEE : 652
OsCRR3            : YMKNGALYDHLHPKPNGSSSPSPSPVATSWKLRIKILLDASRGIDYLHSYAVPPIIHRDIKSSNILLDGS : 620
AtCRR3            : YMKNGALYDHLHDKNNVEKHSS...LINSWKMRIKIALDAARGIEYLHNYAVPPIIHRDIKSSNILLDSN : 642
PHVUL.001G243800  : YMKNGALYDHLHDKNNVEKGSS...VLNYWKMRIKIALDAARGIEYLHNYAVPSIIHRDIKSSNILIDVT : 643
PHVUL.001G243700  : GMKNGSLYDHLHEKG.....SS...VLNSWKMRIKIALDASRGIEYLHKFGVPSPVHGDINPSNILLDAT : 285
PHVUL.001G243500  : YMKNGSLYDHLHHKG.....SV...ELNSWKMRIKIALDASRGIKYLHYYAG.TNIYRDIKSSNILLDDT : 337
PHVUL.001G243600  : YTKNRALYDYLHDKNNVDRESS...ALNSWRMRIKVALDASRGIEYLHKHVVPSIIHRDINSSNILLDAT : 233

                          VII                 VIII                     IX
ZmCR4             : HNARVADFGLSILGPADSGTP....LSELPAGTLGYLDPEYYRLHYLTTKSDVYSFGVVLLEILSGRKAI : 711
ACR4              : HNARVADFGLSLLGPVDSGSP....LAELPAGTLGYLDPEYYRLHYLTTKSDVYSFGVLLLEILSGRKAI : 718
OsCRR3            : WVARVSDFGLSLMGPETEEVK...HLSMKAAGTVGYMDPEYYGLHHLTVKSDVYGFGVVMLEALTGKRAI : 687
AtCRR3            : WVARVSDFGLSLMGPVLGKDHNPYQRPTKAAGTVGYIDPEYYSLNVLTDKSDVYGLGVVLLELLTGKRAI : 712
PHVUL.001G243800  : WTARVSDFGLSLMSPEPDRDY....RPMKAVGTVGYIDPEYYGLNVLTAKSDVYGLGVVLLELLTGKRAI : 709
PHVUL.001G243700  : WTAKVSNIGKAAG................TFGYI...DPEYIDLNVLTTKSDVYGFGVVLLELLTGKN.. : 334
PHVUL.001G243500  : WTARVSDFGLSSL................MKAVGTIIDPEYYGQYVMTAKSEVYAFGVVLLELLTGKR.H : 390
PHVUL.001G243600  : WTARVSGFESSCF................MSPEA...EHVYSDTRVLTAKSDVYGLGVVLLELLTGKKTT : 284

                                            X                         XI
ZmCR4             : DMQFE.........EGNIVEWAVPLIKAGDIFAILDEVLSPPSDL...EALKKIASVACKCVRMRGKDRP : 769
ACR4              : DMHYE.........EGNIVEWAVPLIKAGDINALLDEVLKHPSEI...EALKRIVSVACKCVRMRGKDRP : 776
OsCRR3            : FKEA......EGGSPVSVVDYAVPSIVAGELSKVLDARAPEPSAH.EAEAVELVAYTAVHCVRLEGKDRP : 750
AtCRR3            : FRNNGDVEEEEGCVPVHLVDYSVPAITADELSTILDPRVGSPELG.EGDAVELVAYTAMHCVNAEGRNRP : 781
PHVUL.001G243800  : FKYG.....EDGGTPLSLVDFAVPRILAGEMVKILDPRVGTPDEK.EAEAVELVAYTAIYCVNLEGKDRP : 773
PHVUL.001G243700  : ...........GGT.ILHVPSAEVSILGGDFVKNLDKRVGEPRLN.EAKALKLVAHTAINCVNVEGKVRP : 391
PHVUL.001G243500  : ILCG.....EDGGTPLSVVEFAVPPILDGDLAKILDPRVGAPHVN.EAKAVELMAFTAIHCVNLEGKDRP : 454
PHVUL.001G243600  : LKFG.....INRET..SMVKIAGRVILGWKMVKILDPRVGAPHVNEEAEALEIVAHTAVSCVNSKRKDRP : 347

ZmCR4             : SMDKVTTALEHALALLMGSPCIEQPILPTEVVLGSSRMHKVS.QMSSNHSCSENELADGEDQGIGYRAPS : 838
ACR4              : SMDKVTTALERALAQLMGNPSSEQPILPTEVVLGSSRMHKKSWRIGSKRSGSEN.........TEFRGGS : 837
OsCRR3            : AMADIVANLETAVALCEDSATGGGAAGHGNSSSSASLSITSMELSRMD...................... : 798
AtCRR3            : TMTDIVGNLERALDLCGDSHG.........SISSGICSIVSD............................ : 814
PHVUL.001G243800  : TMADIVVNLERALGICESSHD.........SISSGSISVVSE............................ : 806
PHVUL.001G243700  : TSAQVVLNLERAFAYFRRY................................................... : 410
PHVUL.001G243500  : SLAEIVVNLKRALAIICDSSTHD.......SISNHTIFDVSE............................ : 489
PHVUL.001G243600  : TMTQVVANLETALA.LCDSRPS................................................ : 368

ZmCR4             : WITFPSVTSSQRRKSSASEADIVGRRATDGRNVGSSIGDGLRSLEEEIAPASPQENLYLQHNF : 901
ACR4              : WITFPSVTSSQRRKSSASEGDVAEE.EDEGRKQ....QEALRSLEEEIGPASPGQSLFLHHNF : 895
OsCRR3            : ............................................................... :   -
AtCRR3            : ............................................................... :   -
PHVUL.001G243800  : ............................................................... :   -
PHVUL.001G243700  : ............................................................... :   -
PHVUL.001G243500  : ............................................................... :   -
PHVUL.001G243600  : ............................................................... :   -
```
