# Supplementary material for: Mapping and Genetic Structure Analysis of the Anthracnose Resistance Locus Co-1HY in the Common Bean (Phaseolus vulgaris L.)
Source: PLoS One. 2017 Jan 11;12(1):e0169954. doi: 10.1371/journal.pone.0169954 (PMC5226810; doi:10.1371/journal.pone.0169954)
Supplement: S4 Fig — The black shading indicates polymorphic amino acids. (PDF) [file pone.0169954.s004.pdf]

PHVUL.001G243500

```
Hong_Yundou : MDSESVESIEMWNYGSFDAFSFSLAVDNEIRSGTSSNLGASSPLHSFASLLDIAIR :  56
Jingdou     : MDSESVESIEMWNYGSFDAFSFSLAVDNEIRSGTSSNLGASSPLHSFASLLDIAIR :  56

Hong_Yundou : SRTSSNWETSSANSYAFGLDNAIRSRTSSNLETSSAYSFAFGVDTAIRSRTSSNWE : 112
Jingdou     : SRTSSNWETSSANSYAFGLDNAIRSRTSSNLETSSAYSFAFGVDTAIRSRTSSNWE : 112

Hong Yundou : TSSTHSFAFEEDRTPSNLGSFRVPSFSSGVDYVIRSSSGGTDSETSPVHSFAFAAG : 168
Jingdou     : TSSTHSFAFEEDRTPSNLGSFRVPSFSSGVDYVIRSSSGGTDSETSPVHSFAFAAG : 168

Hong_Yundou : ASQMAANVTGHVAKGLQLFSREELVAATNNFSLHNKIGVGSFGVVYGGLLVDGREV : 224
Jingdou     : ASQMAANVTGHVAKGLQLFSREELVAATNNFSLHNKIGVGSFGVVYGGLLVDGREV : 224

Hong_Yundou : AIKRNETSPKMKEFQEIVFGYLVTFLGRLHHEQLVGLVGFCEEEDEKLLVYEYMKN : 280
Jingdou     : AIKRNETSPKMKEFQEIVFGYLVTFLGRLHHEQLVGLVGFCEEEDEKLLVYEYMKN : 280

Hong Yundou : GSLYDHLHHKGSVELNSWKMRIKIALDASRGIKYLHYYAGTNIYRDIKSSNILLDD : 336
Jingdou     : GSLYDHLHHKGSVELNSWKMRIKIALDASRGIKYLHYYAGTNIYRDIKSSNILLDD : 336

Hong_Yundou : TWTARVSDFGLSSLMKAVGTIIDPEYYGQYVMTAKSEVYAFGVVLLELLTGKRHIL : 392
Jingdou     : TWTARVSDFGLSSLMKAVGTIIDPEYYGQYVMTAKSEVYAFGVVLLELLTGKRHIL : 392

Hong Yundou : CGEDGGTPLSVVEFAVPPILDGDLAKILDPRVGAPHVNEAKAVELMAFTAIHCVNL : 448
Jingdou     : CGEDGGTPLSVVEFAVPPILDGNLAKILDPRVGAPHVNEAKAVELMAFTAIHCVNL : 448

Hong Yundou : EGKDRPSLAEIVVNLKRALAIICDSSTHDSISNHTIFDVSE : 489
Jingdou     : EGKDRPSLAEIVVNLKRALAIICDSSTHDSISNHTIFDVSE : 489
```

PHVUL.001G243600

```
Hong_Yundo : MANNAVDARSFSWAVDSAIASESASNLGESRVHSFASVVDGAIRSSTASEADLEAFPTH :  59
Jingdou    : MANSAVDARSFSWAVDSAIASESASNLGESRVHSFASVVDGAIRSSTASEADLEAFPTH :  59

Hong_Yundo : YFAFASAWDKRTVAARLFTLAELKAATNNFSIHNKIFCAGSISVVHRGKLFDGRQVAVK : 118
Jingdou    : YFAFASAWDKRTVAARLFTLAELKAATNNFSIHNKIFCAGSISVVHRGKLFDGRQVAVK : 118

Hong_Yundo : RAEISSKMKEFQERFGYLWTLLLRLHHKHLVGLVGFCKDKDKRLLVYEYTKNRALYDYL : 177
Jingdou    : RAEISSKMKEFQERFGYLWTLLLRLHHKHLVGLVGFCKDKDKRLLVYEYTKNRALYDHL : 177

Hong_Yundo : HDKNNVDRESSALNSWRMRIKVALDASRGIEYLHKHVVPSIIHRDINSSNILLDATWTA : 236
Jingdou    : HDKNNVDRESSALNSWRMRIKVALDASRGIEYLHKHVVPSIIHRDINSSNILLDATWTA : 236

Hong_Yundo : RVSGFESSCFMSPEAEHVYSDTRVLTAKSDVYGLGVVLLELLTGKKTTLKFGINRETSM : 295
Jingdou    : RVSGFESSCFMSPEAEHVYSDTRVLTAKSDVYGLGVVLLELLTGKKTTLKFGINRETSM : 295

                                                                  *
Hong_Yundo : VKIAGRVILGWKMVKILDPRVGAPHVNEEAEALEIVAHTAVSCVNSKRKDRPTMTQVVA : 354
Jingdou    : VKIAGRVILGWKMVKILDPRVGAPHVNEEAEALEIVAHTAVSCINSKRKDRPTMTQVVA : 354

Hong_Yundo : NLETALALCDSRPS : 368
Jingdou    : NLETALALCDSRPS : 368
```

PHVUL.001G243700

```
Hong_Yundou : MANSGASLVHSFASIVEYAIRSIGGDLGAFPLRDFSFAVENAWSSIGGDLGAVPVR :  56
Jingdou     : MANSGASLVHSFASIVEYAIRSIGGDLGAFPLRDFSFAVENAWSSIGGDLGAVPGR :  56

Hong_Yundou : GFASIVENMMRSVGGDLRALPVHGFASAVENAMISIGGDLGVSPMQGFASRVENAM : 112
Jingdou     : GFASIVENMMRSVGGDLRALPVHGFASAVENAMISIGGDLGVSPMQGFASRVENAM : 112

Hong Yundou : SSIGGDLGTSHSPVHGFQVFTLLELAAATNNFSVDNKIRAGSSSVVYRGKLVDGSE : 168
Jingdou     : SSIGGDLGISHSPVLGFQVFTLLELAAATNNFSVDNKIRAGSSSVVYRGKLVDGSE : 168

Hong_Yundou : VTIERVERWSSRTVEEAFWWRRTSSLKILPGLRPKNLVGLVGLCEEKNERVLVYEG : 224
Jingdou     : VTIERVERWSSRTVEEAFWWRRTSSLKFLPGLRPKNLVGLVGLCEEKNERVLVYEG : 224

Hong_Yundou : MKNGSLYDHLHEKGSSVLNSWKMRIKIALDASRGIEYLHKFGVPSPVHGDINPSNI : 280
Jingdou     : MKNGSLYDHLHEKGSSVLNSWKMRIKIALDASRGIEYLHKYGVPSPVHGDINPSNI : 280

Hong Yundou : LLDATWTAKVSNIGKAAGTFGYIDPEYIDLNVLTTKSDVYGFGVVLLELLTGKNGG : 336
Jingdou     : LLDATWTAKVSNIGKAAGTFGYIDPEYIDLNVLTTKSDVYGFGAVLLELLTGKNGG : 336

Hong_Yundou : TILHVPSAEVSILGGDFVKNLDKRVGEPRLNEAKALKLVAHTAINCVNVEGKVRPT : 392
Jingdou     : TILHVPSAEVSILGGDFVKNLDKRVGEPRLNEAKALKLVAHTAINCVNVEGKVRPT : 392

Hong Yundou : SAQVVLNLERAFAYFRRY : 410
Jingdou     : SAQVVLNLERAIAYFR-Y : 409
```

PHVUL.001G243800

```
Hong_Yundou : MKTLTSSSVTLCLVVAALLILSLPRSSHGLGSGATLTISDASSTVCAVVARESTRRIE :  58
Jingdou     : MKTLTSSSVTLCLVVAALLILSLPRSSHGLGSGATLTISDASSTVCAVVARESTRRIE :  58

Hong_Yundou : CYRQGQIASITPNASFSTISGGRNYFCGLRSSNSDLLCWNTSSSFERRRLYNDSSVPL : 116
Jingdou     : CYRQGQIASITPNASFSTISGGRNYFCGLRSSNSDLLCWNTSSSFERRRLYNDSSVPL : 116

Hong Yundou : ENLAVGDTHLCATEVGDGAVKCWRTGDTFQLPSATDKFASISSGTGFSCGILKNSYRV : 174
Jingdou     : ENLAVGDTHLCATEVGDGAVKCWRTGDTFQLPSATDKFASISSGTGFSCGILKNSSRV : 174

Hong_Yundou : RCWGDTSVSDLTERIESVFGNMSMLSLVAGGSHVCGLNSTGFLVCGGNNDSGQRDFPQ : 232
Jingdou     : RCWGDTSVSDLTERIESVFGNMSMLSLVAGGSHVCGLNSTGFLVCGGNNDSGQRDFPQ : 232

Hong Yundou : GGAFEYSGLALGAEHGCAIRGLNGSVVCWGGNGSFSVNNVTEGVSFEVIVSGSNFVCG : 290
Jingdou     : GGAFEYSGLALGAEHGCAIRGLNGSVVCWGGNGSFSVNNVTEGVSFEVIVSGSNFVCG : 290

Hong Yundou : LTTNNLKVVCWGPGWSNSSTFELPFSTVLPGSCVQSSCECGSYLDSQSLCSGSGNICK : 348
Jingdou     : LTTNNLKVVCWGPGWSNSSTFELPFSTVLPGSCVQSSCECGSYLDSQSLCSGSGNICK : 348

Hong_Yundou : PMTCKLQTTAPPPPSLSPPPPSMSPPPPPPPPPSSSRSKTLTNGLLAFAIIGSVGAFA : 406
Jingdou     : PMTCKLQTTAPPPPSLSPPPPSMSPPPPPPPPPSSSRSKTLTNGLLAFAIIGSVGAFA : 406

Hong Yundou : GICTIVYCLWSGVCFGKKKVHSSVQPTITRGSSGSNGGGASNNSNSSISSMIMRQTSM : 464
Jingdou     : GICTIVYCLWSGVCFGKKKVHSSVQPTITRGSSGSNGGGASNNSNSSISSMIMRQTSM : 464

Hong Yundou : IMRRQRSGTSSTKHPDRAEEFTLAELVAATNNFLLENKIGAGSFGVVYKGKLADGREV : 522
Jingdou     : IMRRQRSGTSSTKHPDRAEEFTLAELVAATNNFLLENKIGAGSFGVVYKGKLADGREV : 522

Hong_Yundou : AIKRGETGSKMKKFQEKESAFESELAFLSRLHHKHLVGLVGFCEEKDERLLVYEYMKN : 580
Jingdou     : AIKRGETGPKMKKFQEKESAFESELAFLSRLHHKHLVGLVGFCEEKDERLLVYEYMKN : 580

Hong Yundou : GALYDHLHDKNNVEKGSSVLNYWKMRIKIALDAARGIEYLHNYAVPSIIHRDIKSSNI : 638
Jingdou     : GALYDHLHDKNNVEKGSSVLNYWKMRIKIALDAARGIEYLHNYAVPSIIHRDIKSSNI : 638

Hong Yundou : LIDVTWTARVSDFGLSLMSPEPDRDYRPMKAVGTVGYIDPEYYGLNVLTAKSDVYGLG : 696
Jingdou     : LIDATWTARVSDFGLSLMSPEPDRDYRPMKAVGTVGYIDPEYYGLNVLTAKSDVYGLG : 696

Hong Yundou : VVLLELLTGKRAIFKYGEDGGTPLSLVDFAVPRILAGEMVKILDPRVGTPDEKEAEAV : 754
Jingdou     : VVLLELLTGKRAIFKYGEDGGTPLSLVDFAVPRILAGEMVKILDPRVGPPDEKEAEAV : 754

Hong Yundou : ELVAYTAIYCVNLEGKDRPTMADIVVNLERALGICESSHDSISSGSISVVSE : 806
Jingdou     : ELVAYTAIYCVNLEGKDRPTMADIVVNLERALGICESSHDSISSGSISVVSE : 806
```
