# Supplementary material for: Mapping and Genetic Structure Analysis of the Anthracnose Resistance Locus Co-1HY in the Common Bean (Phaseolus vulgaris L.)
Source: PLoS One. 2017 Jan 11;12(1):e0169954. doi: 10.1371/journal.pone.0169954 (PMC5226810; doi:10.1371/journal.pone.0169954)
Supplement: S5 Fig — Resistance cultivars: a) G19833, b) Kaboon, c) MDRK, d) Honghuayundou, e) Hongyundou. Susceptible cultivars: f) Jingdou and g) Widusa. Rectangle boxes in solid line reveal the efficient SNPs between resistance cultivars and susceptible cultivars while boxes in dotted line present the meaningless SNPs. (PDF) [file pone.0169954.s005.pdf]

```
a    1 :ATGGCGAATTCAGGAGCATCCCTTGTCCATAGTTTTGCTTCGATAGTGGAATATGCTATTAGAAGTAT
b    1 :ATGGCGAATTCAGGAGCATCCCTTGTCCATAGTTTTGCTTCGATAGTGGAATATGCTATTAGAAGTAT
c    1 :ATGGCGAATTCAGGAGCATCCCTTGTCCATAGTTTTGCTTCGATAGTGGAATATGCTATTAGAAGTAT
d    1 :ATGGCGAATTCAGGAGCATCCCTTGTCCATAGTTTTGCTTCGATAGTGGAATATGCTATTAGAAGTAT
e    1 :ATGGCGAATTCAGGAGCATCCCTTGTCCATAGTTTTGCTTCGATAGTGGAATATGCTATTAGAAGTAT
f    1 :ATGGCGAATTCAGGAGCATCCCTTGTCCATAGTTTTGCTTCGATAGTGGAATATGCTATTAGAAGTAT
g    1 :ATGGCGAATTCAGGAGCATCCCTTGTCCATAGTTTTGCTTCGATAGTGGAATATGCTATTAGAAGTAT

a   69 :TGGAGGAGATTTGGGGGCATTTCCTTTGCGTGATTTTTCTTTCGCAGTGGAGAATGCGTGGAGTAGTA
b   69 :TGGAGGAGATTTGGGGGCATTTCCTTTGCGTGATTTTTCTTTCGCAGTGGAGAATGCGTGGAGTAGTA
c   69 :TGGAGGAGATTTGGGGGCATTTCCTTTGCGTGATTTTTCTTTCGCAGTGGAGAATGCGTGGAGTAGTA
d   69 :TGGAGGAGATTTGGGGGCATTTCCTTTGCGTGATTTTTCTTTCGCAGTGGAGAATGCGTGGAGTAGTA
e   69 :TGGAGGAGATTTGGGGGCATTTCCTTTGCGTGATTTTTCTTTCGCAGTGGAGAATGCGTGGAGTAGTA
f   69 :TGGAGGAGATTTGGGGGCATTTCCTTTGCGTGATTTTTCTTTCGCAGTGGAGAATGCGTGGAGTAGTA
g   69 :TGGAGGAGATTTGGGGGCATTTCCTTTGCGTGATTTTTCTTTCGCAGTGGAGAATGCGTGGAGTAGTA

a  137 :TTGGAGGAGATTTGGGAGCAGTACCTGTTCGTGGTTTTGCTTCTATAGTGGAGAATATGATGAGAAGT
b  137 :TTGGAGGAGATTTGGGAGCAGTACCTGTTCGTGGTTTTGCTTCTATAGTGGAGAATATGATGAGAAGT
c  137 :TTGGAGGAGATTTGGGAGCAGTACCTGTTCGTGGTTTTGCTTCTATAGTGGAGAATATGATGAGAAGT
d  137 :TTGGAGGAGATTTGGGAGCAGTACCTGTTCGTGGTTTTGCTTCTATAGTGGAGAATATGATGAGAAGT
e  137 :TTGGAGGAGATTTGGGAGCAGTACCTGTTCGTGGTTTTGCTTCTATAGTGGAGAATATGATGAGAAGT
f  137 :TTGGAGGAGATTTGGGAGCAGTACCTGGTCGTGGTTTTGCTTCTATAGTGGAGAATATGATGAGAAGT
g  137 :TTGGAGGAGATTTGGGAGCAGTACCTGGTCGTGGTTTTGCTTCTATAGTGGAGAATATGATGAGAAGT

a  205 :GTTGGAGGAGATTTGAGAGCATTACCCGTGCATGGTTTTGCTTCGGCAGTGGAGAATGCAATGATAAG
b  205 :GTTGGAGGAGATTTGAGAGCATTACCCGTGCATGGTTTTGCTTCGGCAGTGGAGAATGCAATGATAAG
c  205 :GTTGGAGGAGATTTGAGAGCATTACCCGTGCATGGTTTTGCTTCGGCAGTGGAGAATGCAATGATAAG
d  205 :GTTGGAGGAGATTTGAGAGCATTACCCGTGCATGGTTTTGCTTCGGCAGTGGAGAATGCAATGATAAG
e  205 :GTTGGAGGAGATTTGAGAGCATTACCCGTGCATGGTTTTGCTTCGGCAGTGGAGAATGCAATGATAAG
f  205 :GTTGGAGGAGATTTGAGAGCATTACCCGTGCATGGTTTTGCTTCGGCAGTGGAGAATGCAATGATAAG
g  205 :GTTGGAGGAGATTTGAGAGCATTACCCGTGCATGCTTTTGCTTCGGCAGTGGAGAATGCAATGATAAG

a  273 :TATTGGAGGAGATTTGGGAGTATCACCCATGCAGGGTTTTGCTTCGAGAGTGGAGAATGCTATGAGTA
b  273 :TATTGGAGGAGATTTGGGAGTATCACCCATGCAGGGTTTTGCTTCGAGAGTGGAGAATGCTATGAGTA
c  273 :TATTGGAGGAGATTTGGGAGTATCACCCATGCAGGGTTTTGCTTCGAGAGTGGAGAATGCTATGAGTA
d  273 :TATTGGAGGAGATTTGGGAGTATCACCCATGCAGGGTTTTGCTTCGAGAGTGGAGAATGCTATGAGTA
e  273 :TATTGGAGGAGATTTGGGAGTATCACCCATGCAGGGTTTTGCTTCGAGAGTGGAGAATGCTATGAGTA
f  273 :TATTGGAGGAGATTTGGGAGTATCACCCATGCAGGGTTTTGCTTCGAGAGTGGAGAATGCTATGAGTA
g  273 :TATTGGAGGAGATTTGGGAGTATCACCCATGCAGGGTTTTGCTTCGAGAGTGGAGAATGCTATGAGTA

a  341 :GCATTGGTGGAGATTTGGGAACATCCCATTCCCCTGTGCATGGTTTTCAAGTATTCACCTTACTTGAG
b  341 :GCATTGGTGGAGATTTGGGAACATCCCATTCCCCTGTGCATGGTTTTCAAGTATTCACCTTACTTGAG
c  341 :GCATTGGTGGAGATTTGGGAACATCCCATTCCCCTGTGCATGGTTTTCAAGTATTCACCTTACTTGAG
d  341 :GCATTGGTGGAGATTTGGGAACATCCCATTCCCCTGTGCATGGTTTTCAAGTATTCACCTTACTTGAG
e  341 :GCATTGGTGGAGATTTGGGAACATCCCATTCCCCTGTGCATGGTTTTCAAGTATTCACCTTACTTGAG
f  341 :GCATTGGTGGAGATTTGGGAATATCCCATTCCCCTGTGCTTGGTTTTCAAGTATTCACCTTACTTGAG
g  341 :GCATTGGTGGAGATTTGGGAATATCCCATTCCCCTGTGCTTGGTTTTCAAGTATTCACCTTACTTGAG

a  409 :CTTGCAGCAGCCACCAACAATTTCTCAGTTGACAACAAGATTCGCGCTGGAAGCTCTAGTGTTGTGTA
b  409 :CTTGCAGCAGCCACCAACAATTTCTCAGTTGACAACAAGATTCGCGCTGGAAGCTCTAGTGTTGTGTA
c  409 :CTTGCAGCAGCCACCAACAATTTCTCAGTTGACAACAAGATTCGCGCTGGAAGCTCTAGTGTTGTGTA
d  409 :CTTGCAGCAGCCACCAACAATTTCTCAGTTGACAACAAGATTCGCGCTGGAAGCTCTAGTGTTGTGTA
e  409 :CTTGCAGCAGCCACCAACAATTTCTCAGTTGACAACAAGATTCGCGCTGGAAGCTCTAGTGTTGTGTA
f  409 :CTTGCAGCAGCCACCAACAATTTCTCAGTTGACAACAAGATTCGCGCTGGAAGCTCTAGTGTTGTGTA
g  409 :CTTGCAGCAGCCACCAACAATTTCTCAGTTGACAACAAGATTCGCGCTGGAAGCTCTAGTGTTGTGTA
```

```
a  477 :CAGGGGAAAACTCGTTGATGGTAGTGAGGTTACAATAGAAAGAGTAGAAAGGTGGAGCAGTAGAACGG
b  477 :CAGGGGAAAACTCGTTGATGGTAGTGAGGTTACAATAGAAAGAGTAGAAAGGTGGAGCAGTAGAACGG
c  477 :CAGGGGAAAACTCGTTGATGGTAGTGAGGTTACAATAGAAAGAGTAGAAAGGTGGAGCAGTAGAACGG
d  477 :CAGGGGAAAACTCGTTGATGGTAGTGAGGTTACAATAGAAAGAGTAGAAAGGTGGAGCAGTAGAACGG
e  477 :CAGGGGAAAACTCGTTGATGGTAGTGAGGTTACAATAGAAAGAGTAGAAAGGTGGAGCAGTAGAACGG
f  477 :CAGGGGAAAACTCGTTGATGGTAGTGAGGTTACAATAGAAAGAGTAGAAAGGTGGAGCAGTAGAACGG
g  477 :CAGGGGAAAACTCGTTGATGGTAGTGAGGTTACAATAGAAAGAGTAGAAAGGTGGAGCAGTAGAACGG

a  545 :TGGAAGAGGCCTTCTGGTGGAGGAGAACGTCTAGTTTGAAGATTTTGCCCGGTTTACGTCCCAAGAAC
b  545 :TGGAAGAGGCCTTCTGGTGGAGGAGAACGTCTAGTTTGAAGATTTTGCCCGGTTTACGTCCCAAGAAC
c  545 :TGGAAGAGGCCTTCTGGTGGAGGAGAACGTCTAGTTTGAAGATTTTGCCCGGTTTACGTCCCAAGAAC
d  545 :TGGAAGAGGCCTTCTGGTGGAGGAGAACGTCTAGTTTGAAGATTTTGCCCGGTTTACGTCCCAAGAAC
e  545 :TGGAAGAGGCCTTCTGGTGGAGGAGAACGTCTAGTTTGAAGATTTTGCCCGGTTTACGTCCCAAGAAC
f  545 :TGGAAGAGGCCTTCTGGTGGAGGAGAACGTCTAGTTTGAAGTTTTTGCCTGGTTTACGTCCCAAGAAC
g  545 :TGGAAGAGGCCTTCTGGTGGAGGAGAACGTCTAGTTTGAAGTTTTTGCCCGGTTTACGTCCCAAGAAC

a  613 :TTGGTTGGGCTGGTTGGGTTGTGTGAGGAGAAAAATGAAAGGGTGTTGGTGTATGAGGGCATGAAGAA
b  613 :TTGGTTGGGCTGGTTGGGTTGTGTGAGGAGAAAAATGAAAGGGTGTTGGTGTATGAGGGCATGAAGAA
c  613 :TTGGTTGGGCTGGTTGGGTTGTGTGAGGAGAAAAATGAAAGGGTGTTGGTGTATGAGGGCATGAAGAA
d  613 :TTGGTTGGGCTGGTTGGGTTGTGTGAGGAGAAAAATGAAAGGGTGTTGGTGTATGAGGGCATGAAGAA
e  613 :TTGGTTGGGCTGGTTGGGTTGTGTGAGGAGAAAAATGAAAGGGTGTTGGTGTATGAGGGCATGAAGAA
f  613 :TTGGTTGGGCTGGTTGGGTTGTGTGAGGAGAAAAATGAAAGGGTGTTGGTGTATGAGGGCATGAAGAA
g  613 :TTGGTTGGGCTGGTTGGGTTGTGTGAGGAGAAAAATGAAAGGGTGTTGGTGTATGAGGGCATGAAGAA

a  681 :TGGGTCATTGTATGATCATTTACATGAGAAGGGTAGCAGTGTGTTGAATTCGTGGAAAATGAGGATAA
b  681 :TGGGTCATTGTATGATCATTTACATGAGAAGGGTAGCAGTGTGTTGAATTCGTGGAAAATGAGGATAA
c  681 :TGGGTCATTGTATGATCATTTACATGAGAAGGGTAGCAGTGTGTTGAATTCGTGGAAAATGAGGATAA
d  681 :TGGGTCATTGTATGATCATTTACATGAGAAGGGTAGCAGTGTGTTGAATTCGTGGAAAATGAGGATAA
e  681 :TGGGTCATTGTATGATCATTTACATGAGAAGGGTAGCAGTGTGTTGAATTCGTGGAAAATGAGGATAA
f  681 :TGGGTCATTGTATGATCATTTACACGAGAAGGGTAGCAGTGTGTTGAATTCGTGGAAAATGAGGATAA
g  681 :TGGGTCATTGTATGATCATTTACGTGAGAAGGGTAGCAGTGTGTTGAATTCGTGGAAAATGAGGATAA

a  749 :AAATTGCTTTGGATGCTTCCCGAGGAATAGAATATCTGCATAAGTTTGGAGTTCCATCTCCTGTTCAT
b  749 :AAATTGCTTTGGATGCTTCCCGAGGAATAGAATATCTGCATAAGTTTGGAGTTCCATCTCCTGTTCAT
c  749 :AAATTGCTTTGGATGCTTCCCGAGGAATAGAATATCTGCATAAGTTTGGAGTTCCATCTCCTGTTCAT
d  749 :AAATTGCTTTGGATGCTTCCCGAGGAATAGAATATCTGCATAAGTTTGGAGTTCCATCTCCTGTTCAT
e  749 :AAATTGCTTTGGATGCTTCCCGAGGAATAGAATATCTGCATAAGTTTGGAGTTCCATCTCCTGTTCAT
f  749 :AAATTGCTTTGGATGCTTCTCGAGGAATAGAATATCTGCATAAGTATGGAGTTCCATCTCCTGTTCAT
g  749 :GAATTGCTTTGGATGCTTCTCGAGGAATAGGATATCTGCATAAGTATGGAGTTCCATCTCCTGTTCAT

a  817 :GGAGATATCAACCCTTCCAACATTCTTCTTGATGCTACTTGGACAGCAAAGGTATCTAACATTGGGAA
b  817 :GGAGATATCAACCCTTCCAACATTCTTCTTGATGCTACTTGGACAGCAAAGGTATCTAACATTGGGAA
c  817 :GGAGATATCAACCCTTCCAACATTCTTCTTGATGCTACTTGGACAGCAAAGGTATCTAACATTGGGAA
d  817 :GGAGATATCAACCCTTCCAACATTCTTCTTGATGCTACTTGGACAGCAAAGGTATCTAACATTGGGAA
e  817 :GGAGATATCAACCCTTCCAACATTCTTCTTGATGCTACTTGGACAGCAAAGGTATCTAACATTGGGAA
f  817 :GGAGATATCAACCCTTCCAACATTCTTCTTGATGCTACTTGGACAGCAAAGGTATCTAACATTGGGAA
g  817 :GGAGATATCAACCCTTCCAACATTCTTCTTGATGCTACTTGGACAGCAAAGGTATCTAACATTGGGAA

a  885 :GGCAGCAGGAACGTTTGGATACATTGATCCTGAGTACATTGATCTGAATGTGTTGACAACAAAGAGTG
b  885 :GGCAGCAGGAACGTTTGGATACATTGATCCTGAGTACATTGATCTGAATGTGTTGACAACAAAGAGTG
c  885 :GGCAGCAGGAACGTTTGGATACATTGATCCTGAGTACATTGATCTGAATGTGTTGACAACAAAGAGTG
d  885 :GGCAGCAGGAACGTTTGGATACATTGATCCTGAGTACATTGATCTGAATGTGTTGACAACAAAGAGTG
e  885 :GGCAGCAGGAACGTTTGGATACATTGATCCTGAGTACATTGATCTGAATGTGTTGACAACAAAGAGTG
f  885 :GGCAGCAGGAACGTTTGGATACATTGATCCTGAGTACATTGATCTGAATGTGTTGACAACAAAGAGTG
g  885 :GGCAGCAGGAACGTTTGGATACATTGATCCTGAGTACATTGATCTGAATGTGTTGACAACAAAGAGTG
```

```
a  953 :ATGTGTATGGATTTGGAGTTGTACTGCTTGAACTTTTAACAGGAAAAAATGGAGGCACCATATTACAT
b  953 :ATGTGTATGGATTTGGAGTTGTACTGCTTGAACTTTTAACAGGAAAAAATGGAGGCACCATATTACAT
c  953 :ATGTGTATGGATTTGGAGTTGTACTGCTTGAACTTTTAACAGGAAAAAATGGAGGCACCATATTACAT
d  953 :ATGTGTATGGATTTGGAGTTGTACTGCTTGAACTTTTAACAGGAAAAAATGGAGGCACCATATTACAT
e  953 :ATGTGTATGGATTTGGAGTTGTACTGCTTGAACTTTTAACAGGAAAAAATGGAGGCACCATATTACAT
f  953 :ATGTGTATGGATTTGGAGCTGTACTGCTTGAACTTTTAACAGGAAAAAATGGAGGCACCATATTACAT
g  953 :ATGTGTATGGATTTGGAGTTGTACTGCTTGAACTTTTAACAGGAAAAAATGGAGGCACCATATTACAT

a 1021 :GTACCCTCTGCAGAGGTTAGTATTTTGGGTGGAGATTTTGTGAAAAATTTGGATAAAAGGGTTGGAGA
b 1021 :GTACCCTCTGCAGAGGTTAGTATTTTGGGTGGAGATTTTGTGAAAAATTTGGATAAAAGGGTTGGAGA
c 1021 :GTACCCTCTGCAGAGGTTAGTATTTTGGGTGGAGATTTTGTGAAAAATTTGGATAAAAGGGTTGGAGA
d 1021 :GTACCCTCTGCAGAGGTTAGTATTTTGGGTGGAGATTTTGTGAAAAATTTGGATAAAAGGGTTGGAGA
e 1021 :GTACCCTCTGCAGAGGTTAGTATTTTGGGTGGAGATTTTGTGAAAAATTTGGATAAAAGGGTTGGAGA
f 1021 :GTACCCTCTGCAGAGGTTAGTATTTTGGGTGGAGATTTTGTGAAAAATTTGGATAAAAGGGTTGGAGA
g 1021 :GTACCCTCTGCAGAGGTTAGTATTTTGGGTGGAGATTTTGTGAAAAATTTGGATAAAAGGGTTGGAGA

a 1089 :ACCCCGTCTCAATGAAGCCAAGGCACTGAAGTTAGTGGCCCGTACTGCCATCAATTGTGTAAATGTGG
b 1089 :ACCCCGTCTCAATGAAGCCAAGGCACTGAAGTTAGTGGCCCATACTGCCATCAATTGTGTAAATGTGG
c 1089 :ACCCCGTCTCAATGAAGCCAAGGCACTGAAGTTAGTGGCCCATACTGCCATCAATTGTGTAAATGTGG
d 1089 :ACCCCGTCTCAATGAAGCCAAGGCACTGAAGTTAGTGGCCCATACTGCCATCAATTGTGTAAATGTGG
e 1089 :ACCCCGTCTCAATGAAGCCAAGGCACTGAAGTTAGTGGCCCATACTGCCATCAATTGTGTAAATGTGG
f 1089 :ACCCCGTCTCAATGAAGCCAAGGCACTGAAGTTAGTGGCCCATACTGCCATCAATTGTGTAAATGTGG
g 1089 :ACCCCGTCTCAATGAAGCCAAGGCACTGAAGTTAGTGGCCCATACTGCCATCAATTGTGTAAATGTGG

a 1157 :AAGGAAAGGTTAGACCAACCAGTGCTCAGGTTGTGTTCAATTTGGAGAGGGCTTTTGCTTATTTCCGG
b 1157 :AAGGAAAGGTTAGACCAACCAGTGCTCAGGTTGTGCTCAATTTGGAGAGGGCTTTTGCTTATTTCCGG
c 1157 :AAGGAAAGGTTAGACCAACCAGTGCTCAGGTTGTGCTCAATTTGGAGAGGGCTTTTGCTTATTTCCGG
d 1157 :AAGGAAAGGTTAGACCAACCAGTGCTCAGGTTGTGCTCAATTTGGAGAGGGCTTTTGCTTATTTCCGG
e 1157 :AAGGAAAGGTTAGACCAACCAGTGCTCAGGTTGTGCTCAATTTGGAGAGGGCTTTTGCTTATTTCCGG
f 1157 :AAGGAAAGGTTAGACCAACCAGTGCTCAGGTTGTGCTCAATTTGGAGAGGGCTATTGCTTATTTCCGG
g 1157 :AAGGAAAGGTTAGACCAACCAGTGCTCAGGTTGTGCTCAATTTGGAGAGGGCTTTTGCTTATTTCCGG

a 1225 :CGGCGGTACTAG
b 1225 :CGG---TACTAG
c 1225 :CGG---TACTAG
d 1225 :CGG---TACTAG
e 1225 :CGG---TACTAG
f 1225 :------TACTAG
g 1225 :------TACTAG
```
