# Supplementary material for: Mapping and Genetic Structure Analysis of the Anthracnose Resistance Locus Co-1HY in the Common Bean (Phaseolus vulgaris L.)
Source: PLoS One. 2017 Jan 11;12(1):e0169954. doi: 10.1371/journal.pone.0169954 (PMC5226810; doi:10.1371/journal.pone.0169954)
Supplement: S6 Fig — The following elements are marked: initiation codon ATG (solid-line arrows); TATA box (grey shading); CAAT box (light grey shading); TC-rich repeats (green shading); TCA-element (red shading); TC-rich repeats (olive green shading); BoxW1 (blue shading); skn-1 motif (blue ash shading); and P-box (clear blue shading). (PDF) [file pone.0169954.s006.pdf]

Phvul.001G243500

```
Hong_Yundou : ATTTGCCAATTAGAACACTGAAACTATATATCTATGGAAATATGTATGTTGAAAGCTTTCATT :  63
Jingdou     : ATTTGCCAATTAGAACACTGAAACTATATATCTATGGAAATATGTATGTTGAAAGCTTTCATT :  63

Hong_Yundou : TTCTGCATTATCCCCAGCTACAGGCAAGTTGAAAAGTGAAACACACCAAACTAGCTTTCATTT : 126
Jingdou     : TTCTGCATTATCCCCAGCTACAGGCAAGTTGAAAAGTGAAACACACCAAACTAGCTTTCATTT : 126

Hong_Yundou : TCTGCATTATCCCCAGCCACAGGCAAGTTGAAAAGTGAAAAACACCAAACTAGCTTCAAACGT : 189
Jingdou     : TCTGCATTATCCCCAGCCACAGGCAAGTTGAAAAGTGAAAAACACCAAACTAGCTTCAAACGT : 189

Hong_Yundou : AGTTAACAAACCATTCAACAAAGGCAGGAAGACACAACTCTCTTGAATCATACGATAAGCAAA : 252
Jingdou     : AGTTAACAAACCATTCAACAAAGGCAGGAAGACACAACTCTCTTGAATCATACGATAAGCAAA : 252

Hong_Yundou : GATATAAAGGACAAAAATGGGTATTCACTAAAATAGTCCCTGGTCCTTTCAAAGATGTTCACA : 315
Jingdou     : GATATAAAGGACAAAAATGGGTATTCACTAAAATAGTCCCTGGTCCTTTCAAAGATGTCCACA : 315

Hong_Yundou : TATTGATGGGGATTTTAATCAAAGACTAAAACATATGGGGATTTAAATAATATACCATTGGAA : 378
Jingdou     : TATTGATGGGGATTTTAATCAAAGACTAAAACATATGGGGATTTAAATAATATACCATTGGAA : 378

Hong_Yundou : ATTTATTATTACATTAATATAAAACAAAATTAGTATAAAGTTTTGTAAATATCTATCATAATA : 441
Jingdou     : ATTTATTATTACATTAATATAAAACAAAATTAGTATAAAGTTTTGTAAATATCTATCATAATA : 441

Hong_Yundou : TGATGCAATATATTATAATATGATGCAATATATTTTAAAAATGTATTATTAAGTCAATAGTCA : 504
Jingdou     : TGATGCAATATATTATAATATGATGCAATATATTTTAAAAATGTATTATTAAGTCAATAGTCA : 504

Hong_Yundou : AATCAATATATTATAATTTAGGATTTATTTATAGAAAATTTTTTCATTGGTATAATAGGTTTT : 567
Jingdou     : AATCAATATATTATAATTTAGGATTTATTTATAGAAAATTTTTTCATTGGTATAATAGGTTTT : 567

Hong_Yundou : CATCTAGTCTTTTTCATCTAGTCTGTTCTAAGAATTTTTTATTAATAGGTATAGTCTACATAT : 630
Jingdou     : CATCTAGTCTTTTTCATCTAGTCTGTTCTAAGAATTTTTTATTAATAGGTATAGTCTACATAT : 630

Hong_Yundou : GTATCGTTTTTGTTAACATATGAGTTTTAGTCTAGTCTTTATATTTTTATTTTTTTTAATAAT : 693
Jingdou     : GTATCGTTTTTGTTAACATATGAGTCTTAGTCTAGTCTCTATATTTTTATTTTTTTTAATAAT : 693

Hong_Yundou : ACTTCTTTTATGTGATGACAAATGATTGTTGTTACTTGAGTTGTCAGCCTAGTTGAGATGTCA : 756
Jingdou     : ACTTCTTTTATGTGATGACAAATGATTGTTGTTACTTGAGTTGTCAGCCTAGTTGAGATGTCA : 756

Hong_Yundou : AGTAGCATAGTGATGTCTAACATGAAAGTTTTTTATAAAAAAAAAATTAAAAAAAAAAGTTAA : 819
Jingdou     : AGTAGCATAGTGATGTCTAACATGAAAGTTTTTTATAAAAAAAAAATTAAAAAAAAAAGTTAA : 819

Hong_Yundou : TATATTATAATATTATGATCTTTATAAAAAAAAAGTCAATATATTATAATATTATG : 875
Jingdou     : TATATTATAATATTATGATCTTTATAAAAAAAAAGTCAATATATTATAATATTATG : 875
```

Phvul.001G243600

```
Hong_Yundou : TACTAGTCAAATTGGAGATTTTTACATAGTTTATAGAGTATAAATCTCATTTTTGTGAGATTG :  63
Jingdou     : TACTAGTCAAATTGGAGATTTTTACATAGTTTATAGAGTATAAATCTCATTTTTGTGAGATTG :  63

Hong_Yundou : AATAAAATGATGGAATTAAAAAATATAGTTAAGTTTTGGGTTGTGTGCTACGTAGCGATAAGA : 126
Jingdou     : AATAAAATGATGGTATTAAAAAAAATAGTTAAGTTTTGGGTTGTGTGCTACGTAGCGATAAGA : 126

Hong Yundou : ATGGTCCCTCATGTAATATTTGATCTTGCAGAAGGATTTTATGGGTATCTTTGAAGTATGAAT : 189
Jingdou     : ATGGTCCCTCATGTAATATTTGATCTTGCAGAAGGATTTTATGGATATCTTTGAAGTATGAAT : 189

Hong_Yundou : TGAATGTTTCACTGTATACGGTACGCGTCAATGAGTGAGGTTGAGTTTCTCTTTTTCCTTGCA : 252
Jingdou     : TGAATGTTTCACTGTATACGCTACGCGTCAATGAGTGAGGTTGAGTTTCTCTTTTTCCTTGCA : 252

Hong_Yundou : GCTTCTCAACTAGTACGCGTTAGATCCCTGCAACTGTGTATATAAGTGTTCTTTAACATAGAT : 315
Jingdou     : GCTTCTCAACTAGTACGCGTTAGATCCCTGCAACTGTGTATATAAGTGTTCTTTAACATAGAT : 315

Hong Yundou : TCATTCATTTAGCTGATGCAGAGTGAAATTTAGCAGAATTGAGATGGCTAATAACGCAGTTGA : 378
Jingdou     : TCATTCATTTAGCTGATGCAGAGTGAAATTTAGCAGAATTGAGATGGCTAATAGCGCAGTTGA : 378

Hong_Yundou : TG : 380
Jingdou     : TG : 380
```

Phvul.001G243700

```
Hong_Yundou : CCCCACCATTACTAACTACACTTCCATTCTACAAAAACACTAAAAAAAAATACTTTTTTT-CTT :  62
Jingdou     : CCCCACCATTACTAACTACACTTCCATTCTACAAAAACACTAAAAAAAAATACTTTTTTTTCTT :  63

Hong_Yundou : CCCGCACCACACCAATTTAGTTGCCGCCACACTAAAAAACTAAATATTATTATTATTGTTATT : 125
Jingdou     : CCTGCACCACACCAATTTAGTTGCCGCCACACTAAAAAACTAAATATTATTATTATTGTTATT : 126

Hong_Yundou : ATTTTATTTATGTAAGATCTTATAATCAATTTGATAAAAAAATTACATTAAAAATTATTAAAT : 188
Jingdou     : ATTTTATTTATGTAAGATCTTATAATCAATTTGATAAAAAAATTACATTAAAAATTATTAAAT : 189

Hong_Yundou : ATCAACTAATTTAAGGATAAAAA-CTACAAATTATTATATTAATTGAGTTATATATTATATTA : 250
Jingdou     : ATCAACTAATTTAAGGATAAAAAACTACAAATTATTATATTAATTAAGTTATATATTATATTA : 252

Hong_Yundou : TCAATCAAAATTTAAAAATAAAATATTATTAATTATTATATTAACTAAGTTATATACTACATT : 313
Jingdou     : TCAATCAAAATTTAAAAATAAAATATTATTAATTATTATATTAACTAAGTTATATACTATATT : 315

Hong_Yundou : RTAAATCAAGATATTAGTATTTAAAGTAGGCTTAATTAGAATCTAAATAGATAGTAACTAAAC : 376
Jingdou     : ATAAATCRAGATATTAGTATTTAAAGTAGGCTTAATTAGAATCTAAATAGATAGTAACTAAAC : 378

Hong_Yundou : CTACTTTAATAGTTTATGACAAATATCGATTTAATTTTTTTTATTAATAATATAAATTAATTT : 439
Jingdou     : CTACTTTAATAGTTTATGACAAATATCAATTTAATTTTTTTTATTAATAATATAAATTAATTT : 441

Hong_Yundou : AGATAATAATAACTTTTTAATTTATAAAATTTATTTAACTTAATTAATATAATGAATAATTAA : 502
Jingdou     : AGATAATAATAACTTTTTAATTTATAAAATTTATTTAACTTAATTAATATAATGAATAATTAT : 504

Hong_Yundou : TTTTT--ATCTTTAAATTAATTGATATTTATTAATTTTGTTTATAGTATACATATATAACTAT : 563
Jingdou     : TTTTTTTATCTTTAAATTAATTGATATTTATTAATTTTGTTTATAGTATGCATATATAAT--- : 564

Hong_Yundou : ATATAAAGTGTGAAATGTAATAAAGTTGAACTATACCAATTAAAATAAAAATATTTGAATTAC : 626
Jingdou     : ----------------------------------------------AAATAT-------AT : 572

Hong_Yundou : AACTATATACCTTTGTAATCTTTTTAACTTGTTAATGTAGCTAAACTATATATAATATTAATG : 689
Jingdou     : AGTTATATACCTTTGTAATCTTTTTAACTTGTTAATGTAACTAAACTATATATAATATTAATG : 635

Hong_Yundou : T---TTTTTTTTAAGTTTTAGTTAAATTCTCAAATATAATTTTTGATGGCAATAATAT----- : 744
Jingdou     : TATTTTTTTTTTAAGTTTTAGTTAAATTCTCAAATATAAATTTTGATGGCAATAATATAATAT : 698

Hong_Yundou : GTTAGCTTTAGTTTGAAGTGACTTATGAAGAAGAAAGTGGTGGAAGGTTACCC---TTCTCTT : 804
Jingdou     : GTTAGCTTTAGTTTGAAGTGACTTATRAAGAGGAAAGTGGTGGAAGGTTACCCCCCTTCTCTT : 761

Hong_Yundou : GCCTCGCACCTTCTTAAAGTGAGTGTTTCAGAAAAATAGGAGAAAAAACGATGGAGCTTTGCA : 867
Jingdou     : GCCTCGCACCTTCTTAAAGTGAGTGTTTCAGAGAAATAGGAGAAAAAACGATGGAGCTTTGCA : 824
```

```
Hong_Yundou : CTCTCTTTGTTTCCATTGTT------------------------------------------- :  887
Jingdou     : CTCTCTTTGTTTCCATTGTTTTATTGTGGATGAAGAGGGATTTAAGAAAACTCAGTCAACCAC :  887

Hong_Yundou : --------------------------------------------------------------- :    -
Jingdou     : AATGAAAGAATAAAAAAACTGTTTGATTTTGAAGGATTGTAAATTGAATAATCGATTATAAAA :  950

Hong_Yundou : -------------ACTATGCTTAAAAATAAATTACACGTGTGTTAAATATAAATATATATAAT :  937
Jingdou     : ATATATATAATTTACTATGCTTAAAAATAAATTGCACGTGTGTTAAATATAAATATA-ATAAT : 1012

Hong_Yundou : AAAAAA-----------------TAAATTTATAATTAAACAAT-ATGAAAAAATATTAAAATG :  982
Jingdou     : AAAAAAATTGTAATTAAACAATATGAAAAAATATTAAAATAATTATGARAAAATATTAAAATG : 1075

Hong_Yundou : ATTAGGAAAAAATATTGAAATTATAGTATTCGGGGGGTTGTAATGTATATAAAAATTCTAAAA : 1045
Jingdou     : ATTAGGAAAAAATATTGAAATAATAGTATTCGGGGG-TTGTAATGTATATAAAAATTCTAA-- : 1135

Hong_Yundou : GTTATGATTAATGGGAATGGCTCTGAAGCACTTTGGCAGAAAAACGAGAATGTATTGCTTTTC : 1108
Jingdou     : GTTATGATTAATGGGAATGGCTCTGAAGCACTTTGGCAGAAAAACGATAATGCATTGCTTTTC : 1198

Hong_Yundou : TTCAGTTACAAATGCAATGGCAGTGCTCTTTTTATAGTCAAATCAYCCACCGCCTATTTTCAA : 1171
Jingdou     : TTCAGTTACAAATGCAATGGCAGTGATCTTTTTATAGTCAAATCATCCACCGCCTATTTTCAA : 1261

Hong_Yundou : AGTCGGTATTTTTAAGATAATTTTGTACCTGTCTTGGACTTTTCACTCATTTGTTTTCGGGGA : 1234
Jingdou     : AGTCGGTATTCTAAAGATAATTTTGTACCTATCTTGGACTTTTCACTCATTTGTTTTCGGGGA : 1324

Hong_Yundou : AATCATGCATACACATGCACTGTTGTTTCTTTTCTTAGTACTTTCTTCTAAAAATTTGTAGCA : 1297
Jingdou     : AATCATGCATACATATGCACTTTTGTTTCTTTTCTTAGTACTTTCTTCTACAAATTTGTAGCA : 1387

Hong_Yundou : ACTGTATATATATGTGTGTCCAAAGCGAGCATGCAGTAGATTCACAAAGCAAAACATTTGGAT : 1360
Jingdou     : ACTATATGTATATATATGTCCAAAGCGAGCATGCAGTAGATTCACAAAGCAAAACATTTGGAT : 1450

Hong_Yundou : TCGCTACACACATAGTTTCACAGTTGAGGCAAGTGGGAAAACAAAAA--------GATTGAAC : 1415
Jingdou     : TCGCTACACACATAGTTTCACAGTTGAGGCAAGTGGGAAAACAAAAACACATTGAGATTGAAC : 1513

Hong_Yundou : TTTCAAGCATATATG : 1430
Jingdou     : TTTCAAGCATATATG : 1528
```

Phvul.001G243800

```
Hong_Yundou : TTTCCCAATCTGGAACATCTTTTTTTAAATTATTCCTATACGTATAGAAAAAAAATATTCATT :  63
Jingdou     : TTTCCCAATCTGGAACATCTTTTTTTAAATTATTCCTATACGTATAGAAAAAAAATATTCATT :  63

Hong_Yundou : TACACGGCACATAAAATAAACCCCATTTACCAATATTTTTAAAGAGGATTCAGTTGAAAATAA : 126
Jingdou     : TACACGGCACATAAAATAAACCCCATTTACCAATATTTTTAAAGAGGATTCAGTTGAAAATAA : 126

Hong_Yundou : TAAAGTGAAAAATAAAAAATAAAAGTTCTAATATGCGTAATGAAACACGTGCAAAGATTATAT : 189
Jingdou     : TAAAGTGAAAAATAAAAAATAAAAGTTCTAATATGCGTAATGAAACACGTGCAAAGATTATAT : 189

Hong_Yundou : ATTCATTTTAAGCATTGATTTTGTTTTAAAAAATGAAAATAAGTAGGATTAGTGTTGTCTGTT : 252
Jingdou     : ATTCATTTTAAGCATTGATTTTGTTTTAAAAAATGAAAATAAGTAGGATTAGTGTTGTCTGTT : 252

Hong_Yundou : TACACTATAATCAGCTTTAGCACTAAATTTTGAGCCCTTAAGTGTACTTATTTGACTCCTCCT : 315
Jingdou     : TACACTATAATCAGCTTTAGCACTAAATTTTGAGCCCTTAAGTGTACTTATTTGACTCCTCCT : 315

Hong_Yundou : CATCAAGATTTGGTCTTCAACCCAAATTTTAAGTTGCCCACTGGGAAAGTACCCACCTCTAAT : 378
Jingdou     : CATCAAGATTTGGTCTTCAACCCAAATTTTAAGTTGCCCACTGGGAAAGTACCCACCTCTAAT : 378

Hong_Yundou : TTTCTTCCTACTTTTTTTTTTATAATTAAAATGTGAAATAAACATATAATAATAGATATTATT : 441
Jingdou     : TTTCTTCCTACTTTTTTTTT-ATAATTAAAATGTGAAATAAACATATAATAATAGATATTATT : 440

Hong_Yundou : TGTTCTAAAGAGAAGGTCTAACAAAATCATTTAATATGTTCTCTTTTTAATTAACATGGTTGT : 504
Jingdou     : TGTTCTAAAGAGAAGGTCTAACAAAATCATTTAATATGTTCTCTTTTTAATTAACATGGTTGT : 503

Hong_Yundou : TCTATTTTTAGTTTCTATTCACTGTATTAGTTATATTTCATTAAATTTTTTATTCGTATTAAA : 567
Jingdou     : TCTATTTTTAGTTTCTATTCACTGTATTAGTTATATTTCATTAAATTTTTTATTCGTATTAAA : 566

Hong_Yundou : GACAATTTAGATGTCTTAATACATCATGAGAATTTACCTATTAATTAGGTTTGATTATTAGTT : 630
Jingdou     : GACAATTTAGATGTCTTAATACATCATGAGAATTTACCTATTAATTAGGTTTGATTATTAGTT : 629

Hong_Yundou : GGATTTTACCTTAAAATCAATCCCAACATATCAATAATACTAATGAATCATTCCTAAATCCAT : 693
Jingdou     : GGATTTTACCTTAAAATCAATCCCAACATATCAATAATACTAATGAATCATTCCTAAATCCAT : 692

Hong_Yundou : AGAACCTGCTCATGATGTCACACTATTTAGTTTCATCTTTATGATATAAAAAAAAATTAAATT : 756
Jingdou     : AGAACCTGCTCATGATGTCACACTATTTAGTTTCATCTTTATGATATAAAAAAAAATTAAATT : 755

Hong_Yundou : GATTGAATAAAAAAAAAAAAATATATATATAT--CTAATTTTTTTAAAATTAAAGATATATAT : 817
Jingdou     : GATTGAATAAAAAAAAATATATATATATATATATCTAATTTTTTTAAAATTAAAGATATATAT : 818

Hong_Yundou : ATATATAATTATTTTTTATAAAATTTATTTAATAAATAATCAAGCTTTTGATATATTTATTAT : 880
Jingdou     : ATA----ATTATTTTTTATAAAATTTATTTAATAAATAATCAAGCTTTTGATATATTTATTAT : 877
```

```
Hong_Yundou : TGGATTAAAACGAAAATGAAAACAAAATTTATATTTATTAAATATTAAATCATGAATAAATAT :  943
Jingdou     : TGGATTAAAACGAAAATGAAAACAAAATTTATATTTATTAAATATTAAATCATGAATAAATAT :  940

Hong_Yundou : AAATTTTTAGGTTATGAACGAAGATAATTATAGTAGAACTGGTATCTGCGACCAAACAGTGCA : 1006
Jingdou     : AAATTTTTAGGTTATGAACGAAGATAATTATAGTAGAACTGGTATCTGCGACCAAACAGTGCA : 1003

Hong_Yundou : AAGAATTGGTCGTTTGGTTTGACCGTCATTGTGAGTCTGGGTCACCCTTTTGAATCATTGCTA : 1069
Jingdou     : AAGAATTGGTCGTTTGGTTTGACCGTCATTGTGAGTCTGGGTCACCCTTTTGAATCATTGCTA : 1066

Hong_Yundou : TCGGTTTAGAAGATTCACTTTAATTCTAATTAAATTATCGATAGCTTAAAACAGGACATTAAT : 1132
Jingdou     : TCGGTTTAGAAGATTCACTTTAATTCTAATTAAATTATCGATAGCTTAAAACAGGACATTAAT : 1129

Hong_Yundou : TAAATAATTGAGTTCAGGTACCAAATTTGAAAATCCAAATTACAACATAGGATTTAAAAATTA : 1195
Jingdou     : TAAATAATTGAGTTCAGGTACCAAATTTGAAAATCCAAATTACAACATAGGATTTAAAAATTA : 1192

Hong_Yundou : AATTAAATAAATACCCGCAGCCTTTTCTTACGTTTTATGATTACGGTCAAACTCTGATTATTA : 1258
Jingdou     : AATTAAATAAATACCCGCAGCCTTTACTTACGTTTTATGATTACGGTCAAACTCTGATTATTA : 1255

Hong_Yundou : TTAAACCCATCTCTTCCACGCTGCTTCGTCCCATCAGAGACACGTGGACCTTCTCCTACTAAA : 1321
Jingdou     : TTAAACCCATCTCTTCCACGCTGCTTCGTCCCATCAGAGACACGTGGACCTTCTCCTACTAAA : 1318

Hong_Yundou : TATTAATATTAAATTTAACACCATAAATTCACGACTTCAAAAAATACTTTTAATCTTAATACG : 1384
Jingdou     : TATTAATATTAAATTTAACACCATAAATTCACGACTTCAAAAAATACTTTTAATCTTAATACG : 1381

Hong_Yundou : ATGTAACACATTATTATTTTTTAAAATTA---------------------------------- : 1413
Jingdou     : ATGTAACACATTATTATTTTTTAAAATTATCAAAGTTTAAATAAAATCATAACTCAAAAAGTG : 1444

Hong_Yundou : ------------------CGAAGTGAACTTTAAGCCTAACTCAATCCCATAAAACCGGCTCAT : 1458
Jingdou     : TTAAAAATATGTGTATTACGAAGTGAACTTTAAGCCTAACTCAATCCCATAAAACCGGCTCAT : 1507

Hong_Yundou : GAGGGTGAGGTTTGCATCCACTTATATACTATGAAAGATTCTAATCTCTAGTCGATGTGAGAT : 1521
Jingdou     : GAGGGTGAGGTTTGCATCCACTTATATACTATGAAAGATTCTAATCTCTAGTCGATGTGAGAT : 1570

Hong_Yundou : CTCCAACACACCTCTCACGCCGATGCTACCAATACGTGCATGAATATATATGTTATAGATGGT : 1584
Jingdou     : CTCCAACACACTTCTCACGCCGATGCTACCAATACGTGCATGAATATATATGTTATAGATGGT : 1633

Hong_Yundou : CCAATAACGGTCCAATAGCGGGTGACATGATAAATGCAACAAATATTCGTTAGGATAGGCTCA : 1647
Jingdou     : CCAATAACGGTCCAATAGCGGGTGACATGATAAATGCAACAAATATTCGTTAGGATAGGCTCA : 1696

Hong_Yundou : AAATGACTCTGATACCATGTTATGAAATGAACTTTAAGCCTAACTCAATCTCATAAAACCGGT : 1710
Jingdou     : AAATGACTCTGATACCATGTTATGAAGTGAACTTTAAGCCTAACTCAATCTCATAAAACCGGT : 1759
```

```
Hong_Yundou : TCATGAGGTTGAGGTTTGCACTCACTTATATACAATGAAGATTTTCTAATCTCTAGTCAACGT : 1773
Jingdou     : TCATGAGGTTGAGGTTTGCACTCACTTATATACAATGAAGATTTTCTAATCTCTAGTCAACGT : 1822


Hong_Yundou : AGGATCTCCAACAATATGAAATAATAATACAAGTTAAAATAAATAAAAAATTTGATG : 1830
Jingdou     : AGGATCTCCAACAATATGAAATAATAATACAAGTTAAAATAAATAAAAAATTTGATG : 1879
```
