# Supplementary material for: Mapping and Genetic Structure Analysis of the Anthracnose Resistance Locus Co-1HY in the Common Bean (Phaseolus vulgaris L.)
Source: PLoS One. 2017 Jan 11;12(1):e0169954. doi: 10.1371/journal.pone.0169954 (PMC5226810; doi:10.1371/journal.pone.0169954)
Supplement: S1 Table — (DOCX) [file pone.0169954.s007.docx]

| Primer name |  | Primer sequences (5'- 3)' | Primer name |  | Primer sequences (5'- 3') |
| --- | --- | --- | --- | --- | --- |
| PSSR0179 | F | TAGACTCTGATGGAAACCGT | PSSR0287 | F | CGATGAAGAAGAAACCATGT |
|  | R | ATGTCACCTGGATATTGCTC |  | R | GCACTCTCTAAACATGCACA |
| PSSR0183 | F | AGAAGCCATGTTGAGAGAGA | PSSR0288 | F | ACTTCAACAACCATCCAAAC |
|  | R | TCAGCCAACTCATTACACAA |  | R | ACACGTACAAATGACAACGA |
| PSSR0184 | F | AGAACTTTCTTGCATACCCA | PSSR0290 | F | AAACTTGTGGATTGAGCTGT |
|  | R | ATGCACACTTTTTACTGGCT |  | R | TCTCATTTGTGCAACTGTTC |
| PSSR0191 | F | CTTATGCAGGCTGCTAGTTT | PSSR0295 | F | TCACTGCACAGTAAACCAAG |
|  | R | ACAGTTGTTGTTTTGGGTGT |  | R | ATGTGGCTAACAAAGCTCAT |
| PSSR0198 | F | CCACAATCCAACATTCTTCT | PSSR0296 | F | CCTCCTAAGCCCATAAGAAC |
|  | R | GCACTCAAACCAAAGAAAAC |  | R | CATTCGCAGTGTCGTTAATA |
| PSSR0217 | F | GAAACAGGATAGGATACATAGG | PSSR0300 | F | GGATTTCTTTTGGAAGAAGG |
|  | R | TTTACTCATTCTGCGGGATA |  | R | AGTCTTCCTTGGGTGATTTT |
| PSSR0230 | F | GTTGCAGAAGAAGGATTGAG | PSSR0752 | F | GCCTTGTGGAGTACTACCTG |
|  | R | ACTTCACGCTGACATACACA |  | R | TGAGAAAGTCTGTTTGAGGA |
| PSSR0233 | F | GTTGCAAAGCCTAATGTGAT | PSSR0755 | F | TGCTCTCTTAGGGTTTGTGT |
|  | R | CACACAAGACTGAACCAGTG |  | R | CCACGTGAGTTTTCTTTTTC |
| PSSR0234 | F | ATGGCATTTCCCATGTTAG | PSSR0757 | F | CCCCACTCACTACCACTAAA |
|  | R | CTGAGCAAAAGCTACATCAA |  | R | CATTGAAGCAAAGGAGAAAC |
| PSSR0242 | F | TCCAATATTAGTCCTCCCAA | PSSR0759 | F | AGATGGTTCACACAAAAAGC |
|  | R | TGTCGTTTTCCTGTTTAGGT |  | R | GGCTGCTGACTTCACTAAAT |
| PSSR0243 | F | TTCTGGCCTTGTTGAATATC | PSS0R771 | F | TACAAAACACGGGTCCTTAC |
|  | R | CATGCACAACATTGATTCTC |  | R | CAACACTCAAGAGTCCATGA |
| PSSR0244 | F | AGTGCAGCTGTCTCAATCTT | PSSR0776 | F | AGACAAACAACTCCACATGA |
|  | R | TATGCTTTATTGACGTGCAG |  | R | GACAACCCAATTTTGTAGGT |
| PSSR0253 | F | TCACCAATTGAGCTTCTTCT | PSSR0779 | F | AACTCAACCATCTTGGTAAG |
|  | R | TTGCAGTGTATGCAGTGATT |  | R | TGAAAAGGATCGAACAAGAC |
| PSSR0254 | F | AAAGACTTTGCTGACAAACC | PSSR0781 | F | ATTTCGTAGGTGGTGCATAC |
|  | R | GCAGCAGATCTTAAGAGACC |  | R | TCTTTCTCTCTCCAAACCAA |
| PSSR0255 | F | TGAACCAGTCATGAGAAACA | PSSR0797 | F | GAGGAGCATGTTAAGGTCTG |
|  | R | TCAGCTTTGATGGAGATACC |  | R | CTGTGTTCTCTTTTCAAGGG |
| PSSR0272 | F | GCCACAAGCTCAAAAGTATC | PSSR0816 | F | TAGGGAGTTGGAGCATAGAA |
|  | R | CATTTTGGTGGGTTTAGGTA |  | R | GAGTGATGTTAGGATTGGGA |

**Table S1:** Newly developed genomic SSR markers for the common bean chromosome 1.
