# Supplementary material for: Mapping and Genetic Structure Analysis of the Anthracnose Resistance Locus Co-1HY in the Common Bean (Phaseolus vulgaris L.)
Source: PLoS One. 2017 Jan 11;12(1):e0169954. doi: 10.1371/journal.pone.0169954 (PMC5226810; doi:10.1371/journal.pone.0169954)
Supplement: S2 Table — (DOCX) [file pone.0169954.s008.docx]

Table S2: The primer sequences of amplifying the gDNA, cDNA and promoter sequences of the candidate genes.

| Candidate genes | Primers name |  | Primer sequences ( 5'- 3') | Predicted Length(bp) |
| --- | --- | --- | --- | --- |
| gDNA sequences amplification primers | | | | |
| *Phvul.001G243500* | PvG1 | F | TTCCTATGGCTGAACTAT | 1516 |
|  |  | R | CACTCAGTTTCTGTTGCA |  |
| *Phvul.001G243600* | PvG2 | F | TTAGATCCCTGCAACTGT | 1308 |
|  |  | R | GAGGAGGTATGAGATCGTCT |  |
| *Phvul.001G243700* | PvG3 | F | AACATTTGGATTCGCTAC | 1446 |
|  |  | R | ATGAGGGACCATTCTTAT |  |
| *Phvul.001G243800* | PvG4-1 | F | TCCCTCAAAGTTCAAACC | 1475 |
|  |  | R | TTATTAGAAGCACCTCCC |  |
|  | PvG4-2 | F | CACCGTCAATGTCACCTC | 1439 |
|  |  | R | ATCTTACAGGCACGGAAT |  |
| cDNA sequences amplification primers | | | | |
| *Phvul.001G243500* | PvC1-1 | F | TATCTGATTTTGGTTTGTC | 423 |
|  |  | R | TATGGTTGGAAATGCTAT |  |
|  | PvC1-2 | F | TTCGTGGAAAATGAGGAT | 215 |
|  |  | R | TCACATATTGACCGTAGTA |  |
|  | PvC1-3 | F | TATTCAGCCGGGAAGAGC | 463 |
|  |  | R | GCCGTCCAAGTATCATCAAG |  |
|  | PvC1-4 | F | TGCTTCGCTTCTGGACAT | 562 |
|  |  | R | CTTGGGACTGGTTTCGTT |  |
|  | PvC1-5 | F | TCATTGCACCTTCTCATC | 347 |
|  |  | R | AAAAGCATAAGAATTGGC |  |
| *Phvul.001G243600* | PvC2 | F | TTAGATCCCTGCAACTGT | 1308 |
|  |  | R | GAGGAGGTATGAGATCGTCT |  |
| *Phvul.001G243700* | PvC3 | F | AACATTTGGATTCGCTAC | 1446 |
|  |  | R | ATGAGGGACCATTCTTAT |  |
| *Phvul.001G243800* | PvC4-1 | F | TCCCTCAAAGTTCAAACC | 1475 |
|  |  | R | TTATTAGAAGCACCTCCC |  |
|  | PvC4-2 | F | CACCGTCAATGTCACCTC | 1439 |
|  |  | R | ATCTTACAGGCACGGAAT |  |
| RT-PCR primers | | | | |
| *Phvul.001G243500* | Pv-CRR1 | F | TATACAGGCCAAGAGTTT | 146 |
|  |  | R | TACCCATGATAGCATTTC |  |
| *Phvul.001G243600* | Pv-CRR2 | F | TTAGATCCCTGCAACTGT | 107 |
|  |  | R | TCAACTGCGTTATTAGCC |  |
| *Phvul.001G243700* | Pv-CRR3 | F | CCATTCTTATCGCTACGT | 156 |
|  |  | R | GGGCTTTTGCTTATTTCC |  |
| *Phvul.001G243800* | Pv-CRR4 | F | TGGTAGGCAATAGGCAAGT | 174 |
|  |  | R | CCCGGGAAAGGGGACAAA |  |
| Promoter sequences amplification primers | | | | |
| *Phvul.001G243500* | PvG5 | F | CTAGGCTGACAACTCAAG | 2018 |
|  |  | R | TTGGACTAATTCCTTGCA |  |
| *Phvul.001G243600* | PvG6 | F | GCACTGTCCACTGCCCAACT | 517 |
|  |  | R | GGCCCGTACTGCCATCAATTGTGTA |  |
| *Phvul.001G243700* | PvG7 | F | TGGATACGGGCTGACTTG | 2266 |
|  |  | R | CATGCTCGCTTTGGACAC |  |
| *Phvul.001G243800* | PvG8 | F | TTCCCAATCTGGAACATC | 1926 |
|  |  | R | GGAAAGGAACGTAGTGTC |  |
|  |  |  |  |  |
