# Supplementary material for: Mapping and Genetic Structure Analysis of the Anthracnose Resistance Locus Co-1HY in the Common Bean (Phaseolus vulgaris L.)
Source: PLoS One. 2017 Jan 11;12(1):e0169954. doi: 10.1371/journal.pone.0169954 (PMC5226810; doi:10.1371/journal.pone.0169954)
Supplement: S3 Table — (DOCX) [file pone.0169954.s009.docx]

Table S3: The primer sequences of the PCR markers.

| Primer name | Forward sequence | Reverse sequence | Referred gene |
| --- | --- | --- | --- |
| Clp-N1 | AGAGTCCACTTCGTCATT | TGATTGGATGGAAGTAAC | Phvul.001G243900.1 |
| STK1 | AAAACATTTGGATTCGCTAC | AACTATGGACAAGGGATG | Phvul.001G243700 |
| TF1 | GACAGTCTCCAGGATGGC | TATGCTTAGTCAATGAAGTT | Phvul.001G243400 |
| Plc1 | TACTCCTTAGGTGGTTTG | AAGCAGTATGCCTTTGTT | Phvul.001G243300 |
