# Supplementary material for: Mapping and Genetic Structure Analysis of the Anthracnose Resistance Locus Co-1HY in the Common Bean (Phaseolus vulgaris L.)
Source: PLoS One. 2017 Jan 11;12(1):e0169954. doi: 10.1371/journal.pone.0169954 (PMC5226810; doi:10.1371/journal.pone.0169954)
Supplement: S4 Table — (DOCX) [file pone.0169954.s010.docx]

Table S4: Gene annotation of the target region.

| Prediction of  potential genes | Homologous genes | Gene annotation^a^ | Position^b^ |
| --- | --- | --- | --- |
| *Phvul.001G243500* | AT3G55950 | serine/threonine-protein kinase-like protein CRR3 (Arabidopsis thaliana) | 50,289,347-50,291,144 |
| *Phvul.001G243600* | AT3G55950 | serine/threonine-protein kinase-like protein CRR3 (Arabidopsis thaliana) | 50,293,967-50,295,727 |
| *Phvul.001G243700* | AT3G55950 | serine/threonine-protein kinase-like protein CRR3 (Arabidopsis thaliana) | 50,296,080-50,297,315 |
| *Phvul.001G243800* | AT3G55950 | serine/threonine-protein kinase-like protein CRR3 (Arabidopsis thaliana) | 50,300,459-50,303,474 |

a:Homologous genes were obtained using BlastP (http://blast.ncbi.nlm.nih.gov/Blast.cgi) based on the predicted genes sequences

b: From <http://phytozome.jgi.doe.gov/pz/portal.html#!info?alias=Org_Pvulgaris>
